# Microbial metabolism mediates the deteriorative effects of sedentary behaviour on insulin resistance

**DOI:** 10.1002/ctm2.70348

**Published:** 2025-05-24

**Authors:** Jingmeng Ju, Jialin He, Bingqi Ye, Siqi Li, Jiaqi Zhao, Wanlan Chen, Qi Zhang, Wanying Zhao, Jialu Yang, Ludi Liu, Yi Li, Min Xia, Yan Liu

**Affiliations:** ^1^ Guangdong Provincial Key Laboratory of Food Nutrition and Health Department of Nutrition School of Public Health Sun Yat‐sen University Guangzhou Guangdong P.R. China; ^2^ Guangdong Provincial Key Laboratory of Food Nutrition and Health Department of Statistics and Epidemiology School of Public Health Sun Yat‐sen University Guangzhou Guangdong P.R. China

**Keywords:** gut microbiota, insulin resistance, Mendelian randomisation, sedentary behaviour

## Abstract

**Background:**

Prolonged sedentary time is a strong risk factor for insulin resistance. Recent evidence indicates that gut microbiota may influence the regulation of insulin sensitivity and demonstrates a distinct profile between sedentary and physically active individuals. However, whether and how microbial metabolism mediates the progression of insulin resistance induced by prolonged sedentary time remains unclear.

**Methods:**

560 male participants without hypoglycaemic therapy were included, and insulin resistance was evaluated using the Homeostatic Model Assessment of Insulin Resistance (HOMA‐IR). The gut microbiota was identified through metagenomics, host genetic data were obtained using a genotyping array, and plasma metabolites were quantified by liquid chromatography mass spectrometry.

**Results:**

A panel of 15 sedentary‐related species and 38 sedentary‐associated metabolic capacities accounted for 31.68% and 21.48% of the sedentary time‐related variation in HOMA‐IR, respectively. Specifically, decreased *Roseburia* sp. *CAG:471*, *Intestinibacter bartlettii*, and *Firmicutes bacterium CAG:83*, but increased *Bacteroides xylanisolvens* related to longer sedentary time, were causally linked to the development of insulin resistance. Furthermore, integrative analysis with metabolomics identified reduced L‐citrulline and L‐serine, resulting from a suppression of arginine biosynthesis as key microbial effectors linking longer sedentary time to enhanced insulin resistance.

**Conclusions:**

In summary, our findings provide insights into the mediating role of gut microbiota on the progression of insulin resistance induced by excessive sedentary time, and highlight the possibility of counteracting the detrimental effect of prolonged sedentary time on insulin resistance by microbiota‐modifying interventions.

**Key points:**

- Prolonged sedentary time leads to a depletion of *Roseburia* sp. *CAG:471* and *Firmicutes bacterium CAG:83*, and suppresses arginine biosynthesis.
- Decreased L‐citrulline and L‐serine function as key microbial effectors mediating the adverse effect of sedentary time on insulin sensitivity.
- Targeting gut microbiota holds promise to combat insulin resistance induced by excessive sedentary time.

## Graphical abstract

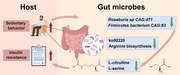

## 1 INTRODUCTION

Insulin resistance, characterised by a reduced response of insulin‐targeting tissues, is a key driver of many metabolic diseases, such as type 2 diabetes, atherosclerotic diseases, and metabolic dysfunction‐associated steatotic liver disease.^1^ Although the underlying mechanism of insulin resistance is not fully understood, driven by economic, social, and technological transitions, recently, excessive sedentary behaviour has been identified as an emerging risk factor.^2^ In adults, time spent sedentary varied from 5 to 11.5 h/day,^3^ and was closely associated with enhanced insulin resistance, even after adjusting for moderate‐to‐vigorous physical activity.^4^ Similarly, a positive association of prolonged uninterrupted sitting with postprandial hyperglycaemia was also found in individuals with either normal or impaired glucose metabolism.^5^ Though the American Diabetes Association had highlighted the importance of interrupting prolonged sedentary periods as a preventive measure against diabetes,^6^ the molecular mechanisms linking sedentary behaviour to metabolic health, including insulin resistance, remains to be elucidated.

Recently, dysbiosis of gut microbiota has been associated with the pathogenesis of insulin resistance through multiple mechanisms. For instance, increased intestinal permeability facilitates bacterial translocation and low grade endotoxemia, which in turn triggers systemic inflammation and impairs insulin signalling. Additionally, perturbations of short‐chain fatty acids (SCFA), secondary bile acids, and branched‐chain amino acids (BCAA) metabolism have been reported to disrupt glucose homeostasis by affecting hepatic glucose production, release of glucagon‐like peptide‐1, and insulin sensitivity in peripheral tissues.^7^, ^8^ Distinct makeup of gut microbiota had been found in individuals with various degrees of insulin resistance^9^ and animal models,^10^ whereas, fecal microbial transplantation from healthy donors was reported to enhance insulin sensitivity in patients with severe obesity and metabolic disorders.^11^ Of note, among the various factors shaping the microbial communities, lifestyle practices, including sedentary behaviour, emerge as the most influential ones.^12^ A remarkable decrease in both diversity and network complexity of gut microbiota was found in sedentary compared to active individuals.^13^ Moreover, the microbiome of professional athletes demonstrated a more favourable metabolic capacity, such as amino acid and antibiotic biosynthesis and carbohydrate metabolism, compared to sedentary counterparts,^14^ further supporting a modulatory role of physical activity in gut microbiota. However, whether and how alterations in gut microbiota are functionally involved in the adverse effect of prolonged sedentary time on insulin resistance remain obscure.

Here, to elucidate how gut microbial alterations mediate the adverse effect of sedentary time on insulin resistance, we conducted an integrative multiomics analysis, including gut microbiota, host genetics, and plasma metabolites in a deeply phenotyped cohort of male participants without hypoglycaemic therapies.

## 2 MATERIALS AND METHODS

### 2.1 Study design and population

South China Cohort (SCC)‐deep was a multiomics subcohort conducted in Dongguan City, Guangdong Province, P.R. China.^15^ Ethical approval for this study was granted by the Ethics Committee of School of Public Health, Sun Yat‐Sen University (2017‐001), and all procedures were performed in accordance with the principles of the Declaration of Helsinki. Written informed consents were obtained from each individual. To exclude the complex effects of hormones on both gut microbiota and insulin sensitivity, only male participants were included in this study. Detailed inclusion and exclusion criteria of SCC‐deep were provided in Supplementary Methods (Additional file 1).

**FIGURE 1:**
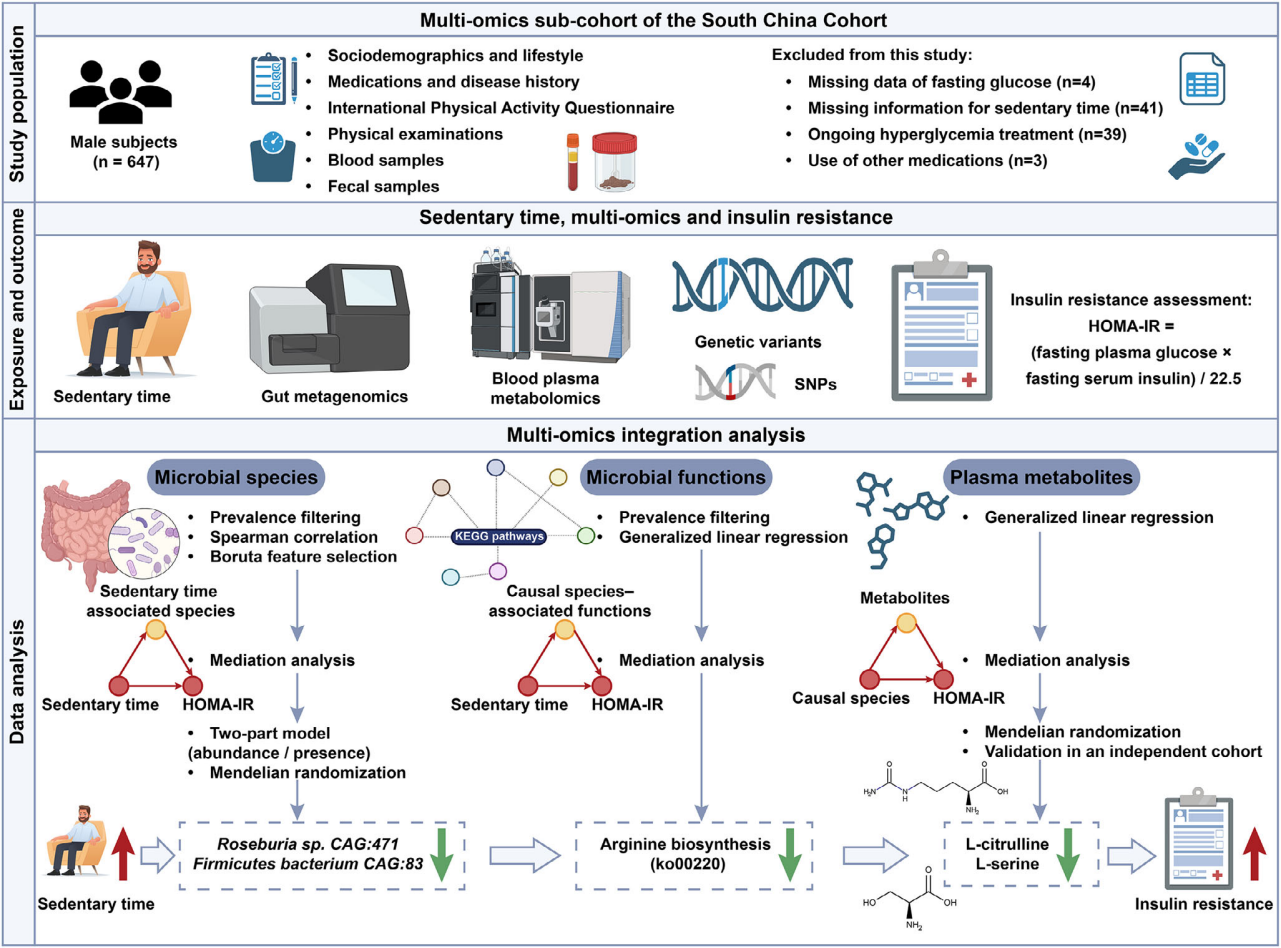
Flowchart of the study. 560 male individuals free of hypoglycaemic treatment and antibiotics use prior to sample collection were included, and subjected to integrative analysis of fecal metagenomic sequencing, genotyping, and plasma metabolomics.

Among the 647 male participants from SCC‐deep, 87 individuals were further excluded based on the following reasons: (1) missing data of fasting glucose (*n* = 4); (2) missing information for sedentary time (*n* = 41); (3) ongoing hyperglycaemia treatment (*n* = 39); (4) use of drugs known to affect gut microbiota (*n* = 3). In total, 560 individuals were included in this analysis (Figure 1). Detailed procedures for data collection were provided in Supplementary Methods (Additional file 1).

### 2.2 Ascertainment of insulin resistance and sedentary time

Homeostatic Model Assessment for Insulin Resistance (HOMA‐IR), calculated as [fasting insulin (pmol/L) × fasting glucose (mmol/L)]/135,^16^ was employed to determine insulin resistance. Individuals were categorised into high or low HOMA‐IR groups based on a threshold of 2.5, as previously described.^17^ Sedentary time was assessed using the International Physical Activity Questionnaire Short Form (IPAQ‐SF), a widely used tool for the quantification of long‐term physical activity and sedentary time in large‐scale population studies. The study participants reported their sedentary time by indicating the total time (in hours and minutes) spent on sitting on a typical day over the past 7 days.^18^ Sedentary time and HOMA‐IR were primarily analysed as continuous variables. Given the lack of a universally accepted threshold of sedentary time, we categorised participants based on the median sedentary time of the study population (6.8 h per day) when group comparisons were conducted, as previously described.^19^

### 2.3 Fecal DNA isolation and sequencing

Fecal samples were collected and processed following a standardised protocol, with DNA extraction and metagenomic sequencing performed on the Illumina NovaSeq 6000 platform. Fecal samples were collected on the same day as blood drawing. More details about sample handling, quality control, and sequencing parameters are available in Supplementary Methods.

### 2.4 Plasma metabolomics profiling

After an overnight fast of at least 10 h, blood samples were collected concurrently with stool collection, and preserved at −80°C until analysis. An established protocol for large‐scale profiling of broadly targeted metabolites was applied, as described in the previous study.^20^ Additional information on metabolomics profiling and downstream analyses were provided in Supplementary Methods.

### 2.5 Human genomics and bidirectional Mendelian randomisation analysis

Host genomic DNA was isolated from the buffy coat using a commercial blood DNA extraction kit, according to the manufacturer's instruction. DNA samples with a concentration ≥80 ng/µL and total yield ≥1 µg were used for library construction. More details about genotyping and quality control were provided in Supplementary Methods. To explore host genetic variants linked to gut microbiota or microbial metabolites, a threshold of *p *< 5×10^−5^ was adopted to maximise explained genetic variance, as previously described.^21^ To investigate the causal associations between microbial species and metabolites on HOMA‐IR variability, bidirectional Mendelian Randomisation (MR) analysis was performed using the *MendelianRandomization* R package (version 0.10.0). MR estimates were evaluated by three methods, including inverse‐variance weighted (IVW), weighted median and MR‐Egger regression, with the IVW method serving as the primary method. More details were provided in Supplementary Methods.

### 2.6 Statistical analysis

The normality of continuous variables was assessed using the Shapiro–Wilk test and Q–Q plots. Data with approximate normal distribution were summarised as mean and standard deviation (SD), while skewed variables were expressed as median with interquartile range (25^th^–75^th^ percentile). The comparison of basic characteristics was performed using the Student's *t*‐test or Wilcoxon rank‐sum test, based on the distribution of the data. Categorical variables were expressed as counts and percentages, and analysed with Chi‐squared test. Missing data were handled by multiple imputation via the *mice* R package. Spearman rank correlation coefficient was calculated using cor.test. To adjust for multiple comparisons, false discovery rate (FDR) correction was applied using 1000 permutations, with statistical significance defined as FDR *p*  <  .05. All analyses were conducted by R software (V4.4.1, R Foundation for Statistical Computing, Vienna, Austria).

## 3 RESULTS

### 3.1 Microbial species mediate the impact of sedentary time on insulin resistance

A total of 560 male participants (median age: 50 years, interquartile range: 44–56) without history of hyperglycaemia treatment or antibiotic use were analysed. Overall, individuals with longer sedentary periods were younger, more likely to have less physical activity, higher education attainment, worse lipid profiles, in addition to poorer glycaemic control (Table 1).

**TABLE 1:** Basic characteristics of the study participants.

	Sedentary time (h/day)			
Characteristics	All (*n* = 560)	Low (≤6.8) (*n* = 280)	High (> 6.8) (*n* = 280)	*p* value
**Demographic and socioeconomic characteristics**				
Age, year	50.00 (44.00, 56.00)	52.00 (45.00, 60.00)	49.00 (43.00, 54.00)	<.001
Education, *n* (%)				
Primary or below	91 (16.52%)	56 (20.36%)	35 (12.70%)	.001
Middle school	426 (77.31%)	211 (76.73%)	215 (77.9%)	
College or above	34 (6.17%)	8 (2.91%)	26 (9.40%)	
Household annual income (Yuan/year), *n* (%)				
< 60 000	120 (21.78%)	72 (26.18%)	48 (17.39%)	.063
60 000–100 000	185 (33.58%)	91 (33.09%)	94 (34.06%)	
100 000–150 000	186 (33.76%)	87 (31.64%)	99 (35.87%)	
≥150 000	60 (10.89%)	25 (9.09%)	35 (12.68%)	
**Physical examinations**				
BMI, kg/m^2^	24.63 ± 3.34	24.29 ± 3.45	24.97 ± 3.20	.017
Waist, cm	88.30 (81.47, 93.93)	87.35 (80.00, 92.53)	89.55 (82.72, 94.50)	.004
SBP, mmHg	125.00 (117.50, 137.00)	125.00 (117.38, 136.50)	126.00 (117.50, 137.50)	.575
DBP, mmHg	83.00 (77.50, 90.00)	82.50 (75.50, 90.00)	83.50 (78.00, 89.62)	.057
**Clinical tests**				
Fasting plasma glucose, mmol/L	4.74 (4.29, 5.24)	4.62 (4.20, 5.20)	4.83 (4.43, 5.24)	.008
Fasting insulin, pmol/L	62.67 (40.16, 104.88)	54.89 (36.95, 88.32)	73.68 (44.99, 124.99)	<.001
HOMA‐IR	2.21 (1.38, 4.07)	1.96 (1.25, 3.18)	2.55 (1.55, 4.61)	<.001
TG, mmol/L	1.33 (0.95, 1.92)	1.22 (0.85, 1.81)	1.41 (1.02, 2.03)	.001
TC, mmol/L	5.18 (4.68, 5.75)	5.10 (4.60, 5.57)	5.36 (4.77, 5.97)	<.001
HDL‐c, mmol/L	1.25 (1.05, 1.43)	1.24 (1.04, 1.44)	1.25 (1.06, 1.42)	.825
LDL‐c, mmol/L	3.17 (2.72, 3.65)	3.12 (2.71, 3.54)	3.21 (2.73, 3.82)	.014
**Lifestyle**				
Smoking, *n* (%)	272 (48.60%)	151 (53.90%)	121 (43.20%)	.014
Drinking, *n* (%)	79 (14.11%)	37 (13.21%)	42 (15.00%)	.627
Diet diversity score	5.00 (4.75, 6.00)	5.00 (4.00, 6.00)	5.00 (5.00, 6.00)	.307
MET‐ h/week	34.65 (13.20, 67.99)	39.33 (15.05, 69.30)	26.95 (11.55, 46.20)	<.001

*Note*: Data were expressed as mean ± SD, median (interquartile range), or *n* (%). *p* values were determined by Student's *t*‐test, Wilcoxon rank‐sum test, Chi‐square test or Fisher exact test, as appropriate. Number of missing variables: Education (*n* = 9), Income (*n* = 9).

Abbreviations: BMI, body mass index; MET, metabolic equivalent; HOMA‐IR, homeostatic model assessment of insulin resistance; SBP, systolic blood pressure; DBP, diastolic blood pressure; TG, triglyceride; TC, total cholesterol; HDL‐c, high‐density lipoprotein cholesterol, and LDL‐c, low‐density lipoprotein cholesterol.

**FIGURE 2:**
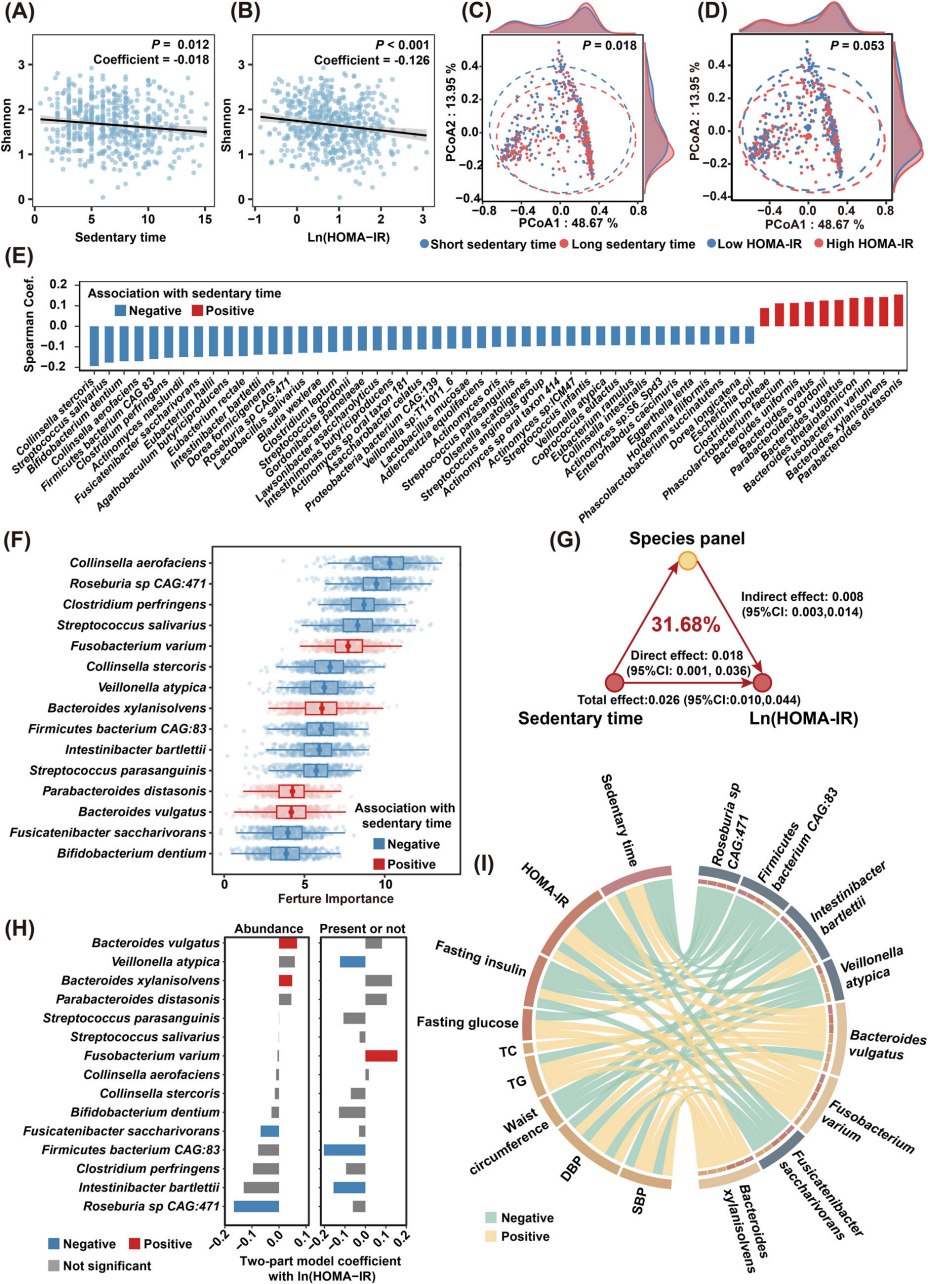
Microbial species partially mediate the effect of sedentary time on insulin resistance. Association of alpha diversity with (A) sedentary time and (B) HOMA‐IR (*n* = 560). The *p*‐values were derived from linear models and were adjusted for age, BMI, smoking, drinking, diet diversity, educational attainment and household income. (C, D) Principal Coordinates Analysis plots of beta diversity of gut microbiota at genus level based on Bray–Curtis distance (*p* calculated by PERMANOVA), stratified by the median value of (C) sedentary time and by a threshold of 2.5 for (D) HOMA‐IR. (E) Species significantly correlated with sedentary time. (F) Boxplots of the normalised permutated variable importance of species associated with sedentary time. Significant species were identified by the random forest‐based machine learning variable selection algorithm Boruta using 1000 trees, 500 iterations, with FDR *p *< .05. Since Boruta only provided feature importance without directions, the directions of the association with sedentary time were determined by the coefficient in E. (G) Estimates of the mediation effect of the species identified in F on the association between sedentary time and HOMA‐IR, adjusted for the same variables in A. (H) Species significantly associated with HOMA‐IR, determined by a two‐part model adjusted for the same variables in A. Blue, red and grey indicated negative, positive and insignificant correlations with HOMA‐IR, respectively. (I) The chord diagram showing the associations between clinical phenotypes and species identified in H, after adjustment for the same covariates in A. Yellow and green indicated positive and negative associations derived from the two‐part model. For clarity, only significant associations with FDR *p* < .05 were shown.

We then explored the potential role of gut microbiota in sedentary time and insulin resistance. At community level, the diversity of gut microbiota was found to be inversely correlated with both sedentary time (*p *< .05, Figure 2A) and insulin resistance (*p *< .05, Figure 2B), after adjustment for potential confounders that might affect gut microbiota, such as age, body mass index (BMI), smoking, drinking, diet diversity, educational attainment and household income. Moreover, a clear segregation in beta diversity was observed in individuals with higher or lower sedentary time (*p *< .05, PERMANOVA, Figure 2C). A similar, albeit marginal, segregation in beta diversity was observed between individuals with high or low HOMA‐IR levels (*p *= .053, PERMANOVA, Figure 2D), suggesting a potential link between sedentary time, gut microbiota alterations, and insulin resistance. Of note, a total of 55 species, mainly from *Bacillota* (formerly *Firmicutes*), *Actinomycetota* and *Bacteroidota* phyla, showed significant associations with sedentary time (FDR *p *< .05, Figure 2E and Table S1). Of the 15 key microbial species closely associated with sedentary time, *Fusobacterium varium*, *Bacteroides xylanisolvens*, *Parabacteroides distasonis*, and *Bacteroides vulgatus* demonstrated a positive association with sedentary time, while the other 11 species showed a negative association (Figure 2F). The 15 species collectively accounted for 31.68% of the sedentary time‐related variation in insulin resistance, after correcting for demographic, lifestyle and socioeconomic factors (*p *< .05, Figure 2G). Among the 15 microbial species associated with sedentary time, only 8 species, the majority of which were well‐known SCFA producers, such as *Intestinibacter bartlettii* (*I. bartlettii*),^22^ *Roseburia sp CAG:471*, *Firmicutes bacterium CAG:83*, *Fusicatenibacter saccharivorans* (*F. saccharivorans*) and *Veillonella atypica* (*V. atypica*), were also significantly associated with HOMA‐IR (Figure 2H). Consistently, all these 5 SCFA‐producers were also found to be associated with better lipid profiles, lower fasting glucose, insulin levels and BMI (Figure 2I). On the contrary, *Bacteroides xylanisolvens* (*B. xylanisolvens*), *Bacteroides vulgatus* (*B. vulgatus*),^23^ and *Fusobacterium varium* (*F. varium*),^24^ which were previously reported to be enriched in insulin‐resistant or obese individuals, demonstrated a positive association with longer sedentary time, higher HOMA‐IR, elevated DBP and worse lipid profiles (Figure 2I). Overall, our findings indicated that microbial species may serve as intermediaries linking sedentary time and insulin resistance.

### 3.2 Causal association between sedentary‐shaped species and the progression of insulin resistance

**FIGURE 3:**
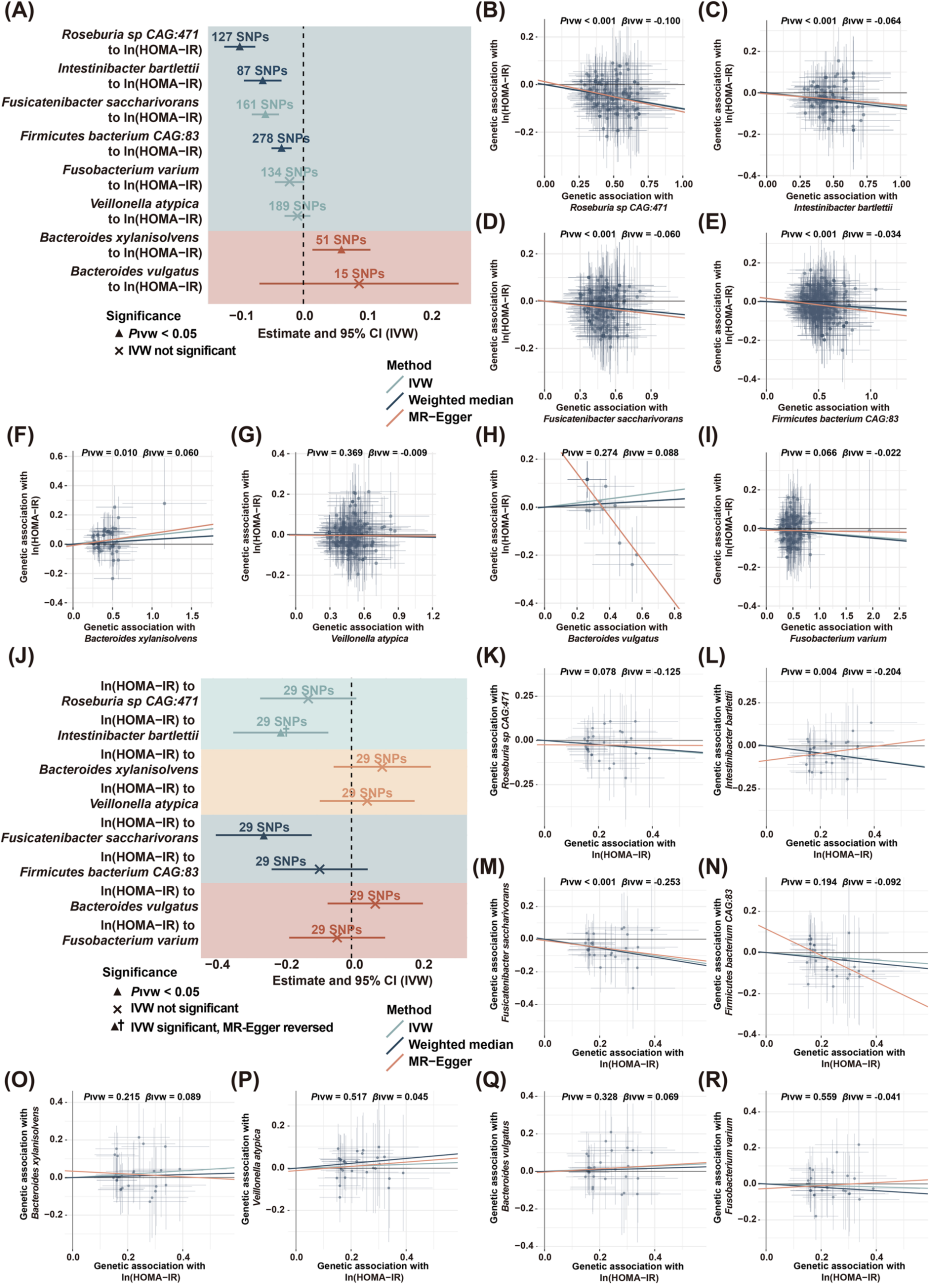
Causal links between selected species and HOMA‐IR. (A) The forest plot showing the potential causal associations observed between selected species and HOMA‐IR (*n* = 554). Data were presented as beta coefficients with corresponding 95% confidence intervals. (B–I) Scatterplot of associations between genetic variants and selected species including (B) *Roseburia sp CAG:471*, (C) *Intestinibacter bartlettii*, (D) *Fusicatenibacter saccharivorans*, (E) *Firmicutes bacterium CAG:83*, (F) *Bacteroides xylanisolvens*, (G) *Veillonella atypica*, (H) *Bacteroides vulgatus* and (I) *Fusobacterium varium* versus between genetic variants and HOMA‐IR. The slope of each line corresponded to the estimated Mendelian Randomisation effect. (J) The forest plot showing the potential causal associations observed between HOMA‐IR and selected species (*n* = 554). (K–R) Scatterplot of associations between genetic variants and HOMA‐IR versus between genetic variants and selected species including (K) *Roseburia sp CAG:471*, (L) *Intestinibacter bartlettii*, (M) *Fusicatenibacter saccharivorans*, (N) *Firmicutes bacterium CAG:83*, (O) *Bacteroides xylanisolvens*, (P) *Veillonella atypica*, (Q) *Bacteroides vulgatus* and (R) *Fusobacterium varium*.

We conducted a bidirectional MR analysis to investigate the causal relationship between sedentary‐associated microbial species and the development of insulin resistance. Only 5 species, including *Roseburia sp CAG:471*, *I. bartlettii*, *F. saccharivorans*, *Firmicutes bacterium CAG:83* and *B. xylanisolvens*, were potentially causally linked to HOMA‐IR (Figure 3A–I). Specifically, an IVW estimate indicated that *Roseburia sp CAG:471* (Beta_IVW _= –0.100, 95% CI = –0.125 to –0.076, *p *< .001), *I. bartlettii* (Beta_IVW _= –0.064, 95% CI = –0.093 to –0.034, *p *< .001), *F. saccharivorans* (Beta_IVW _= –0.060, 95% CI = –0.081 to –0.038, *p *< .001) and *Firmicutes bacterium CAG:83* (Beta_IVW _= –0.034, 95% CI = –0.050 to –0.018, *p *< .001, Figures 3B–E and S1A) enhanced insulin sensitivity. Additionally, these microbial species were negatively associated with longer sedentary time and higher insulin levels (Figure 2I). On the contrary, *B. xylanisolvens* (Beta_IVW _= 0.060, 95% CI = 0.014 to 0.105, *p *= .010) exacerbated insulin resistance (Figures 3F and S1A), and was found to be positively associated with longer sedentary time, higher SBP, DBP, glucose and TG levels (Figure 2I **)**. However, the IVW estimates did not support any causal associations of *V. atypica*, *B. vulgatus* and *F. varium* with HOMA‐IR (Figure 3G–I). Importantly, the directionality of effect estimated from the IVW method were consistent with those derived from the weighted median and MR‐Egger methods, and all instrumental variables (IVs) had *F*‐statistics > 10, effectively eliminating the bias of weak IVs. Moreover, no significant heterogeneity was detected among these IVs based on Cochran's Q test from the IVW analysis. Similarly, the MR‐Egger regression intercept analysis did not indicate any evidence of directional horizontal pleiotropy in the observed causal estimates (Figure S1A). Additionally, sensitivity analysis using a leave‐one‐out approach further demonstrated that the observed causal associations between *Roseburia sp CAG:471*, *I. bartlettii*, *F. saccharivorans*, *Firmicutes bacterium CAG:83* and *B. xylanisolvens* and HOMA‐IR were robust and not disproportionately influenced by any single SNP (Figure S2).

Moreover, reverse MR analysis revealed no significant causal effects of HOMA‐IR on *Roseburia sp CAG:471*, *Firmicutes bacterium CAG:83* and *B. xylanisolvens* in both IVW and weighted median analyses, while the effects on *F. saccharivorans* were significant, indicating a bidirectional effect (Figures 3J–R and S1B). Of note, although the causal effect of HOMA‐IR on *I. bartlettii* was significant in IVW analysis, the direction was opposite to that in the MR‐Egger method (Figures 3L and S1B), suggesting that this causal association was not reliable.^25^ Collectively, the findings demonstrated that *Roseburia sp CAG:471*, *I. bartlettii*, *Firmicutes bacterium CAG:83* were negatively correlated with sedentary time and causally linked to an amelioration of HOMA‐IR, whereas *B. xylanisolvens* was positively associated with sedentary time and causally linked to a progression of insulin resistance.

### 3.3 Sedentary time‐related microbial capacities contribute to the progression of insulin resistance

**FIGURE 4:**
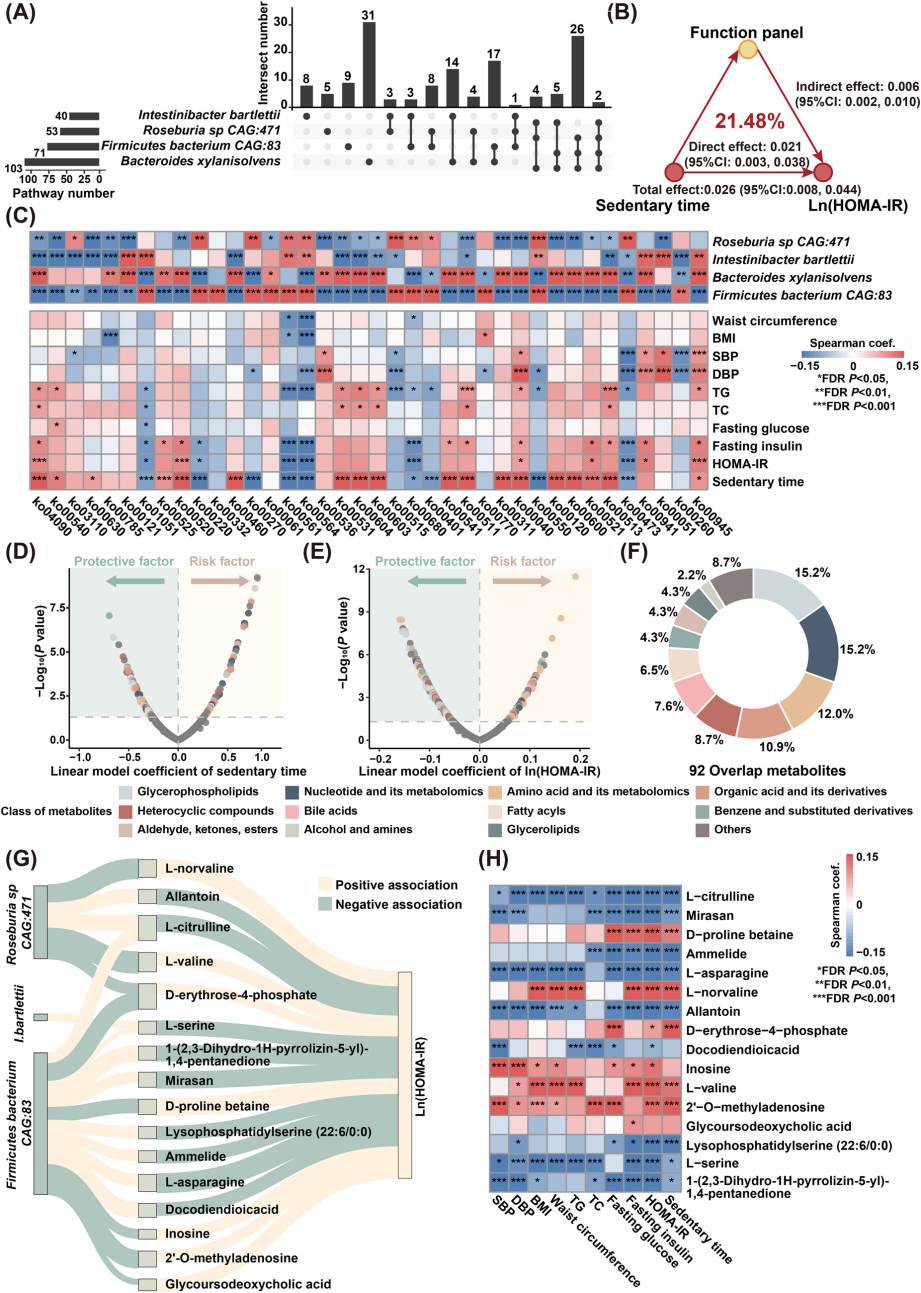
Sedentary‐associated microbial functions and metabolites are closely associated with HOMA‐IR (A) Upset plot showing the overlap of microbial functions significantly associated with selected species (*n* = 560). Generalised linear model was adopted, adjusting for age, BMI, smoking, drinking, diet diversity, educational attainment and household income. (B) Estimates of the mediation effect of selected microbial functions on the association between sedentary time and HOMA‐IR. Mediation analyses were adjusted for the same covariates in A. (C) Heatmap showing the correlation between clinical indices and microbial functions that were significantly associated with at least three species shown in A. For clarity, only microbial functions and clinical indices with at least one significant correlation (^*^FDR *p* < .05, ^**^FDR *p* < .01, ^***^FDR *p* < .001) were shown. Metabolites significantly associated with (D) sedentary time and (E) HOMA‐IR, identified after adjustment for the same covariates in A. FDR *p* < .05 was considered significant. Nodes were coloured based on the Class I classification of metabolites. (F) Pie chart showing the distribution of metabolites that were significantly associated with both sedentary time and HOMA‐IR, with consistent directionality of the associations. (G) Mediation linkages among selected species, plasma metabolites and HOMA‐IR were adjusted for the same covariates as in A, and significance was assessed after FDR correction. Yellow and green lines indicated positive and negative correlations, respectively. The thickness of the lines was proportional to the absolute value of the strength of association. (H) Heatmap showing the correlation between clinical indices and plasma metabolites identified in G.

To further elucidate the mechanisms by which reduced *Roseburia sp CAG:471*, *I. bartlettii*, *Firmicutes bacterium CAG:83*, and increased *B. xylanisolvens* influenced host metabolism and promoted insulin resistance, microbial genes were functionally annotated using Kyoto Encyclopedia of Genes and Genomes (KEGG) database. After adjustment for demographics, lifestyle and socioeconomic factors, a total of 38 metabolic functions, spanning amino acid, fatty acid and glycan metabolism, were found to be significantly associated with at least three of the four pivotal sedentary‐associated species (FDR *p *< .05, Figure 4A and Table S2). Collectively, these microbial functions explained 21.48% of the sedentary time‐related variation in HOMA‐IR (Figure 4B). Of note, almost all the microbial functions demonstrated a consistent direction of correlation with both sedentary time and HOMA‐IR (Figure 4C). More specifically, microbial amino acid biosynthesis, such as arginine biosynthesis (ko00220), and cysteine and methionine metabolism (ko00270), and lipid metabolism, including both phosphatidylcholine biosynthesis (ko00564) and its upstream regulator glycerolipid metabolism (ko00561), were inversely associated with both sedentary time and HOMA‐IR. Additionally, both phosphatidylcholine biosynthesis (ko00564) and glycerolipid metabolism (ko00561) were also negatively associated with adverse lipid profiles and obesity (Figure 4C). By contrast, sphingolipid metabolism (ko00600), particularly ceramide biosynthesis, glycosphingolipid biosynthesis (ko00603, ko00604), pathways involved in bacterial outer membrane components, such as lipopolysaccharide biosynthesis (ko00540), glycosaminoglycan degradation (ko00531) and O‐antigen nucleotide sugar biosynthesis (ko00541) exhibited positive associations with sedentary time. In addition, glycosaminoglycan degradation (ko00531) was positively associated with high glucose levels (Figure 4C). Moreover, functions responsible for glucuronate supply and nucleotide sugar synthesis (ko00040, ko00520) demonstrated a positive association with both sedentary time and insulin resistance (Figure 4C). Taken together, these findings underscored the mediation of the sedentary lifestyle on insulin resistance, through alterations in microbial metabolism.

Given that metabolites often serve as effectors between gut microbiota and host metabolism,^26^ integrated metagenomics‐metabolomics analysis was employed to further explore how sedentary‐related microbial metabolites modulated insulin resistance. After controlling for demographics, lifestyle and socioeconomic factors, 206 metabolites and 407 metabolites were significantly associated with sedentary time and HOMA‐IR, respectively (FDR *p *< .05, Figures 4D and E and S3A and B). Among them, 92 metabolites spanning glycerophospholipids, nucleotide and its derivative, amino acids, organic acid and its derivatives, and heterocyclic compounds and bile acids, demonstrated significant associations with both sedentary time and HOMA‐IR with consistent directionality (Figure 4F). Furthermore, 16 of these metabolites mediated the effect of *Roseburia sp CAG:471*, *I. bartlettii*, and *Firmicutes bacterium CAG:83* on insulin resistance (FDR *p*_mediation _< 0.05), while no obvious mediating link between *B. xylanisolvens* and HOMA‐IR was observed, after controlling for demographics, lifestyle and socioeconomic factors (Figures 4G and S4A–S). Particularly, increased D‐erythrose‐4‐phosphate accounted for 6.59% of the effect of decreased *Roseburia* sp. *CAG:471* and 16.19% of the effect of decreased *Firmicutes bacterium CAG:83*, respectively, on worsening HOMA‐IR (Figure S4E and H). Additionally, elevated L‐norvaline and L‐valine accounted for the largest proportion of the effect of reduced *Roseburia* sp. *CAG:471* on insulin resistance, contributing 18.95% and 15.64%, respectively to the exacerbation of HOMA‐IR (Figures 4G and S4A and D). Consistently, these two metabolites were also found to be positively correlated with worse lipid profiles, and higher waist circumference (Figure 4H). Conversely, reduced allantoin accounted for 12.39% of the effect of decreased *Roseburia* sp. *CAG:471* on the worsening of insulin sensitivity (Figures 4G and S4B), and demonstrated an positive association with adverse metabolic profiles, such as obesity and elevated blood pressure (Figure 4H). In a similar fashion, increased levels of L‐asparagine and lysophosphatidylserine (22:6/0:0) contributed to 21.76% and 16.07% of the effect of *Firmicutes bacterium CAG:83* on improved insulin sensitivity, respectively (Figures 4G and S4O and M). Taken together, these findings highlighted the potential of microbial metabolites as molecular transducers linking prolonged sedentary time to insulin resistance.

### 3.4 L‐citrulline and L‐serine serve as key microbial effectors linking sedentary time to insulin resistance

**FIGURE 5:**
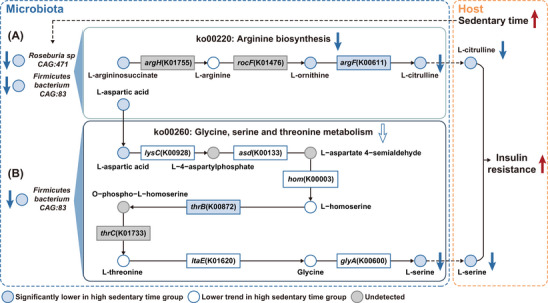
Schematic diagram illustrating how prolonged sedentary time suppresses the microbial production of L‐citrulline and L‐serine. (A, B) Prolonged sedentary time led to a reduction in *Roseburia sp CAG:471* and *Firmicutes bacterium CAG:83*, which encoded key enzymes responsible for (A) arginine biosynthesis and (B) glycine, serine and threonine metabolism (*n* = 560). The downregulation of arginine biosynthesis and glycine, serine and threonine metabolism led to a decreased production of L‐citrulline and L‐serine, which in turn enhanced insulin resistance.

Beyond numerical links observed above, metabolites with significant mediatory effects were further mapped to the KEGG database to explore their biological links. Intriguingly, *argH* and *argF*, key enzymes involved in arginine biosynthesis (ko00220), can be annotated in the genomes of *Roseburia sp CAG:471* and *Firmicutes bacterium CAG:83* through protein sequence BLAST (Figure 5A). In individuals with excessive sedentary time, the abundance of these two species was remarkably lower (Figure S5A and B), leading to a downregulation of arginine biosynthesis (ko00220) (Figure S5C). Such a downregulation was associated with a marked reduction in key intermediate metabolites along this pathway, such as L‐arginosuccinate and L‐ornithine, which in turn led to a suppressed production of L‐citrulline, primarily due to a reduction in *argF* (Figure S5D–G), which can be encoded by both of these two species (Figure 5A). Notably, decreased L‐citrulline accounted for 14.21% of the effect of decreased *Roseburia* sp. *CAG:471* and 19.14% of the effect of decreased *Firmicutes bacterium CAG:83*, respectively on insulin resistance (Figure S4C and G). Consistently, L‐citrulline was found to be substantially lower in individuals with longer sedentary time and demonstrated a negative association with insulin resistance, adverse lipid profiles, high BMI, and elevated blood pressure (Figures 4H and S5H). On the other hand, *asd* and *thrC* can also be annotated in the genome of *Firmicutes bacterium CAG:83* (Figure 5B). Notably, L‐aspartic acid served as a critical intermediate metabolite linking arginine biosynthesis (ko00220) and glycine, serine, and threonine metabolism (ko00260). In individuals with longer sedentary time, L‐aspartic acid levels were found to be remarkably lower, which in turn contributed to a decreasing trend in ko00260 (Figures 5B and S5G–I). Consistent with the pathway‐level inference, most of the key intermediate metabolites and enzymes within this pathway, including *lysC*, *asd*, *hom*, L‐homoserine, L‐threonine, *ltaE*, glycine and *glyA* exhibited a downregulated trend, and *thrB* was significantly lower in subjects with longer sedentary time (Figure S5K–S), which ultimately resulted in a markedly reduced biosynthesis of L‐serine (Figures 5B and S5T). Remarkably, reduced L‐serine accounted for 7.54% of the effect of *Firmicutes bacterium CAG:83* on insulin resistance (Figure S4I), and was inversely associated with HOMA‐IR, unfavourable lipid profiles, central obesity, and elevated blood pressure (Figure 4H).

**FIGURE 6:**
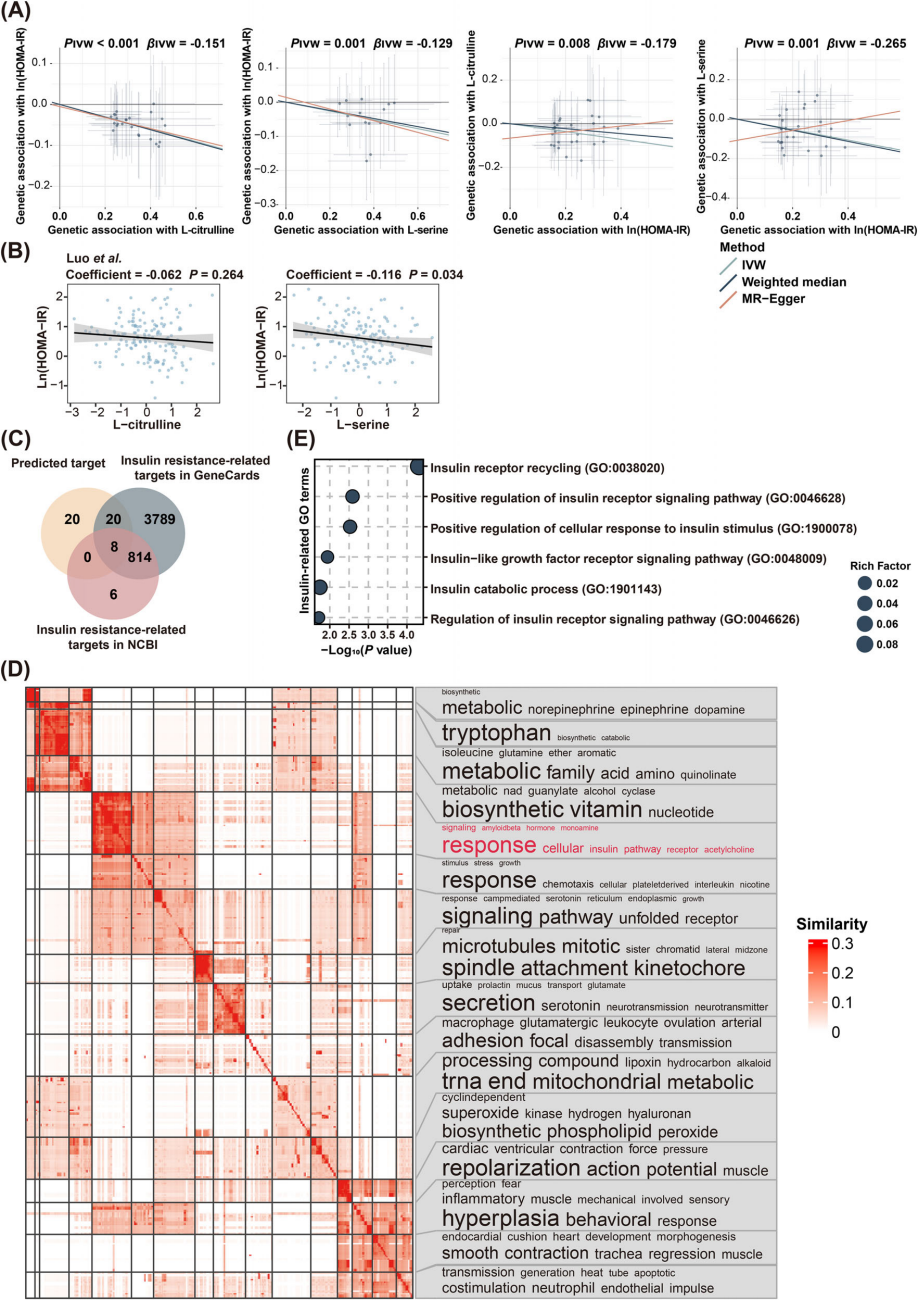
Causal links between L‐citrulline, L‐serine and insulin resistance. (A) Scatterplot of associations between genetic variants and selected metabolites versus between genetic variants and HOMA‐IR (*n* = 554). The slope of each line corresponded to the estimated Mendelian Randomisation effect. (B) Associations of the abundance of L‐citrulline and L‐serine with HOMA‐IR in an independent validation cohort. (C) Overlap of potential downstream targets of L‐citrulline and L‐serine with genes related to insulin resistance in National Center for Biotechnology Information and GeneCards repositories. (D) Similarity clustering heatmap for enriched pathways with term frequencies exhibited by font size in biological processes. (E) Significantly enriched Gene Ontology terms related to insulin signalling pathway.

Furthermore, bidirectional MR analysis demonstrated that both L‐citrulline and L‐serine were causally linked to the variation of HOMA‐IR, whereas, no significant effects of HOMA‐IR on these two metabolites were found (Figure 6A). Moreover, all IVs had *F*‐statistics > 10, eliminating the risk of weak IV bias. Additionally, no significant heterogeneity was detected among these IVs based on Cochran's Q test from the IVW analysis. Similarly, the MR‐Egger regression intercept analysis indicated no evidence of directional horizontal pleiotropy in these causal estimates (Figure S6A). Additionally, sensitivity analysis using a leave‐one‐out approach further demonstrated that the observed causal associations between L‐citrulline and L‐serine and HOMA‐IR were robust and not disproportionately influenced by any single SNP (Figure S6B), suggesting that L‐citrulline and L‐serine were causally linked to an amelioration of insulin resistance. More importantly, the inverse associations between L‐citrulline, L‐serine, and HOMA‐IR were further validated in an independent validation cohort^27^ (Figure 6B), lending further support to the crucial role of L‐citrulline and L‐serine in the alleviation of insulin resistance induced by prolonged sedentary time. Furthermore, more than half of the downstream targets of L‐citrulline and L‐serine had been previously linked to the development of insulin resistance (Figure 6C). For instance, the nitric oxide synthase (NOS) family of enzymes, including NOS2 and NOS3,^28^ were found to be potential downstream targets of L‐citrulline by in silico analysis. Consistent with our finding, increased NOS2 expression in the skeletal muscle of obese mice had been reported to be associated with enhanced S‐nitrosation of key insulin signalling components, such as the insulin receptor, insulin receptor substrate 1, and protein kinase B, which in turn disrupted insulin signalling and promoted insulin resistance.^29^, ^30^ In contrast, NOS3 deficiency had been found to be associated with reduced fatty acid oxidation,^31^ elevated circulating triglyceride levels, and fasting hyperinsulinemia.^32^ Similarly, nuclear factor kappa‐B, a downstream target of both L‐citrulline and L‐serine, had been proved to be a key player in obesity‐induced low‐grade inflammation and insulin resistance.^33^ Gene Ontology (GO) enrichment revealed that the downstream targets of L‐citrulline and L‐serine were primarily linked to metabolic processes and various signalling responses (Figure 6D). Notably, several GO terms associated with insulin receptor signalling or cellular response to insulin stimulus were identified (Figure 6E), supporting that reduced *Roseburia* sp. *CAG:471* and *Firmicutes bacterium CAG:83* due to excessive sedentary time may impair insulin signalling and promote insulin resistance. Overall, these findings indicated that decreased levels of L‐citrulline and L‐serine functioned as critical microbial effectors linking the detrimental effect of sedentary behaviour on insulin resistance.

## 4 DISCUSSION

Although prior small‐scale human studies have suggested a distinct microbial composition between sedentary and physically active individuals,^13^, ^14^ and a potential mediating role of gut microbes in insulin sensitivity,^17^, ^23^ the contribution of microbial metabolism to sedentary behaviour–related insulin resistance remains poorly understood. To our knowledge, this is the first study to dissect the underlying mechanism whereby prolonged sedentary time shapes the composition and metabolic functions of gut microbiota in humans, offering additional insights into how microbial metabolism functions as molecular transducers linking unhealthy lifestyle to adverse metabolic health. We first demonstrated that microbial species and metabolic capacities accounted for 31.68% and 21.48% of the sedentary time‐related variation in HOMA‐IR, respectively. More specifically, reduced *I. bartlettii*, *Roseburia* sp. *CAG:471*, and *Firmicutes bacterium CAG:83*, but increased *B. xylanisolvens* were causally linked to the deterioration of insulin resistance induced by prolonged sedentary time. Additionally, through integrated analysis of multiomics and MR, reduced production of L‐citrulline and L‐serine, resulting from suppressed arginine biosynthesis of gut microbes, were identified as key molecular transducers connecting excessive sedentary time to the development of insulin resistance.

Though how prolonged sedentary time affects gut microbiota remains unclear and warrants further investigation, emerging evidence suggest that lifestyle factors, such as diet, exercise, and sleep disturbance, may contribute to the remodelling of gut microbiota by amplifying the subtle difference in intestinal microenvironment, including oxidative status and local immunity, which are critical for the growth, colonisation and interactions of the gut microbes.^34^, ^35^ Of note, despite several pieces of evidence linking physical activity or exercise and alterations of gut microbiota, sedentary behaviour and physical inactivity are conceptually distinct, rather than simply opposite or compensatory states. Prolonged sedentary time was reported to be independently associated with adverse health outcomes even in individuals who met the guideline‐recommended physical activity.^36^ Recently, emerging evidence implies that disturbed composition and metabolic capacities of the gut microbiota may serve as molecular transducers linking excessive sedentary time to insulin resistance. Similar to our observation, studies in free‐living individuals from both Spain^13^ and Sweden^37^ identified a negative association between microbial diversity and sedentary time. More specifically, consistent with the negative associations of *I. bartlettii*, *Roseburia* sp. *CAG:471*, and *Firmicutes bacterium CAG:83* with sedentary time, a higher abundance of *I. bartlettii*,^37^ *Roseburia* spp.,^37^ and *Firmicutes* ^38^ was also found in individuals with higher levels of physical activity from Europe, further suggesting that these species may function as universal biomarkers for inactivity. Consistent with a decreased abundance of *B. xylanisolvens* in prediabetic individuals with improved insulin sensitivity following exercise intervention,^34^ a positive association of *B. xylanisolvens* with both sedentary time and HOMA‐IR was also found in our study. More importantly, decreased abundance of these species were found in individuals suffering from various metabolic disorders closely associated with insulin resistance. For instance, consistent with the negative association of *I. bartlettii* and *Roseburia* sp. *CAG:471* with insulin resistance observed in our study, a decreased abundance of *I. bartlettii* and *Roseburia* genus was reported in postmenopausal women with obesity^39^ and patients with type 2 diabetes.^40^ In a similar fashion, the negative association of *Firmicutes bacterium CAG:83* and hepatic steatosis,^41^ a key feature of hepatic insulin resistance, further supported the inverse association between *Firmicutes bacterium CAG:83* and insulin resistance observed in this study. Importantly, leveraging the causal inference framework of the bidirectional MR analysis, we provided additional evidence by showing that *I. bartlettii*, *Roseburia sp CAG:471*, *Firmicutes bacterium CAG:83* and *B. xylanisolvens* were causally associated with HOMA‐IR. Though further investigation in preclinical models and more diverse populations are warranted, our study helps bridge the knowledge gap by providing multiomics evidence linking sedentary behaviour to gut microbial composition and its downstream metabolic consequences. As a major driver of cardiometabolic disorders, our findings suggested that gut microbiota‐modulating interventions might provide additional benefits to reduce the adverse effect of excessive sedentary time on insulin resistance.

Unlike previous studies using 16S rRNA sequencing, which lacks functional resolution,^42^ this study benefits from the integration of metagenomics and metabolomics, we found that microbial lipid metabolism played a key regulatory role in the effect of prolonged sedentary time on insulin resistance. Consistent with a suppression of glycerophospholipid metabolism in individuals with a higher sedentary time, lysophosphatidylserine (22:6/0:0), a signalling phospholipid mainly responsible for the resolution of inflammation,^43^ was found to be markedly reduced in individuals with high sedentary time and enhanced insulin resistance. Of note, lysophosphatidylserine (22:6/0:0) alone accounted for 16.07% of the total effect of *Firmicutes bacterium CAG:83* on insulin resistance. On the contrary, prolonged sedentary time was linked to an enhanced biosynthesis of sphingolipids (ko00600) and glycosphingolipids (ko00603, ko00604), which in turn led to insulin resistance. This finding was further supported by a previous report that sphingolipid metabolism was activated in patients with gestational diabetes mellitus.^44^ Mechanistically, sphingolipids play a crucial role in glucolipotoxicity‐induced apoptosis, contributing to the progression of insulin resistance.^45^ In addition to lipid metabolism, a disturbance in glucose utilisation, another important metabolic capacity of gut microbiota, was also found in individuals with excessive sedentary time. In line with an upregulation of pentose phosphate metabolism (ko00030) and pentose and glucuronate interconversions (ko00040), D‐erythrose‐4‐phosphate, a key intermediate of pentose phosphate metabolism, was remarkably higher in individuals with insulin resistance. Importantly, as a precursor in shikimate metabolism, D‐erythrose‐4‐phosphate initiated the biosynthesis of aromatic amino acids in gut microbiota,^46^ the increased level of which was implicated in the impairment of insulin sensitivity in various murine models.^47^ Moreover, similar to an elevation in microbial capacities for glycosaminoglycan degradation in individuals with excessive sedentary time in Poland,^48^ a positive association between glycosaminoglycan degradation (ko00531) and sedentary time was also observed in our study. Additionally, given the well‐established link between lipopolysaccharides from gram‐negative bacteria and metabolic disorders,^49^ it was not surprising to find an enhanced microbial capacity responsible for bacterial outer membrane synthesis, such as lipopolysaccharide biosynthesis (ko00540) and O‐antigen nucleotide sugar biosynthesis (ko00541), in subjects with a prolonged sedentary time. Importantly, the positive association of the gram‐negative bacterium *B. xylanisolvens* with these pathways observed further supported its role in the exacerbation of insulin resistance induced by excessive sedentary time.

Furthermore, integrative analysis of metagenomics and metabolomics indicated that amino acid metabolism in the gut microbiota played a pivotal regulatory effect of sedentary behaviour on insulin resistance. Consistent with the independent association of baseline BCAAs levels with increased risk of insulin resistance and type 2 diabetes observed in the Framingham Offspring Study,^50^ enhanced microbial production of L‐valine, which was reported to induce insulin resistance via the disruption of lipid oxidation in skeletal muscle,^51^ was also found in individuals with high sedentary time and HOMA‐IR in our study. This was further supported by a recent study showing that L‐valine supplementation in lean mice induced gut inflammation, enhanced adipogenesis, and disrupted lipid metabolism, highlighting its potential role in metabolic dysfunction.^52^ Consistent with our findings that L‐asparagine levels were negatively associated with sedentary time and HOMA‐IR, higher plasma L‐asparagine was reported to be significantly associated with improved insulin sensitivity in Finnish men, indicating a potential protective role of L‐asparagine in metabolic homeostasis.^53^ On the contrary, in accordance with a negative association of allantoin with both sedentary time and HOMA‐IR observed in our study, its deficiency has been reported in patients with gestational diabetes mellitus,^54^ while allantoin itself was found to downregulate glucotoxicity and lipotoxicity in type 2 diabetic rats.^55^ Additionally, chronic administration of allantoin was found to reduce body weight, epididymal fat mass, and energy intake in mice challenged with high‐fat diet, suggesting a potential antiobesity effect.^56^ Moreover, decreased arginine biosynthesis (ko00220) of the gut microbiota in individuals with a prolonged sedentary time led to a lower abundance of L‐citrulline in the circulation, which in turn promoted insulin resistance. In collaboration with our observation, supplementation of L‐citrulline was found to be effective in the amelioration of insulin resistance in obese individuals with type 2 diabetes.^57^ Aligned with the in silico findings that the downstream targets of L‐citrulline were enriched in the insulin signalling pathway, L‐citrulline was reported to enhance hepatic insulin sensitivity via reducing the phosphorylation of insulin receptor substrate‐1 at serine 1101.^58^ Similarly, in line with a decreased capacity for arginine biosynthesis (ko00220) and a downward trend in glycine, serine, and threonine metabolism (ko00260) in individuals with excessive sedentary time, lower level of L‐serine was found in subjects with high sedentary time and HOMA‐IR. This finding was further supported by previous studies linking higher L‐serine levels with enhanced insulin sensitivity in individuals diagnosed with nonalcoholic fatty liver disease.^59^ Mechanistically, increased L‐serine enhanced intracellular NAD+ content and led to the activation of mitochondria biogenesis and fatty acid oxidation, which in turn reversed insulin resistance in peripheral tissues.^60^ More importantly, in accordance with the mediation analysis showing that reduced L‐citrulline and L‐serine accounted for around 7.54∼19.14% of the effect of decreased *Roseburia* sp. *CAG:471* and *Firmicutes bacterium CAG:83*, the key enzymes involved in L‐citrulline and L‐serine production can be annotated in these two sedentary‐associated species. Taken together, the above findings underscored the potential of L‐citrulline and L‐serine as microbial effectors linking excessive sedentary time and insulin resistance, and highlighted the potential of developing gut microbiota‐targeted strategies to mitigate the adverse effect of high sedentary time on metabolic health, especially in individuals with objectively prolonged sedentary time due to clinical conditions, such as paralysis, joint disorders, and neuromuscular diseases. Through an integration of multiomics analysis, L‐citrulline and L‐serine were identified as two postbiotic metabolites that could combat the detrimental effect of prolonged sedentary time on insulin resistance. Compared to fecal microbiota transplantation and probiotics, postbiotic‐based intervention serves as a more direct and safer therapeutic alternative^61^ for individuals with limited mobility or metabolic disorders. To further validate the potential of L‐citrulline and L‐serine supplementation in the amelioration of insulin resistance, more investigation in diverse populations, preclinical models, and clinical trials are warranted.

Despite comprehensive adjustments were made for potential confounders, certain limitations of the study remain unavoidable. First, sedentary behaviour was self‐reported, which was inevitable for recall bias. Nonetheless, the international physical activity questionnaire has been widely accepted and validated in various large‐scale cohorts.^18^ Second, the cross‐sectional design limited causal inference. Although the integration of multiomics and MR analyses strengthened the robustness of our findings, future longitudinal studies are warranted to further validate the causal relationships observed in this study. Third, the study was conducted exclusively in Chinese males, the current findings may not be directly applicable to females. Given the remarkable impact of sex and menopause status on gut microbiota and the considerable effect of menopause status on insulin resistance, in depth studies specifically in female populations should be performed to figure out the role of gut microbiota in the association between sedentary time and insulin resistance. Though the associations of L‐citrulline and L‐serine, two pivotal microbial effectors, with insulin sensitivity have been validated in an independent cohort, future studies with larger sample sizes from more geographically and ethnically diverse populations are warranted. Such studies would further strengthen the generalisability of our findings across different genetic backgrounds and environmental exposures. Fourth, despite an FDR correction, the risk of false positives inherent to large‐scale multiomics association analyses remains. However, the rigorous integration of multiomics data strengthens the biological relevance and interpretation of our findings. Nevertheless, more studies in preclinical models should be conducted in the future to further delineate the role of sedentary behaviour in microbial production of L‐citrulline and L‐serine.

## 5 CONCLUSION

In summary, our study uncovers a crucial regulatory role of microbial metabolism in the effect of excessive sedentary time on insulin resistance and identifies L‐citrulline and L‐serine as key molecular transducers linking sedentary time and insulin resistance. Our results underscore the promise of targeting gut microbiota to mitigate the adverse metabolic effects of prolonged sedentary time.

## AUTHOR CONTRIBUTIONS

Yan Liu and Min Xia conceived and designed this study. Jingmeng Ju, Yan Liu, and Min Xia drafted and revised the manuscript for intellectual content. Jialin He, Bingqi Ye, and Ludi Liu established the platform for omics data analysis. Jingmeng Ju carried out the analysis. Siqi Li, Jiaqi Zhao, Wanlan Chen, Qi Zhang and Wanying Zhao helped with the recruitment of study participants and sample collection. Jialu Yang and Yi Li helped with the processing of biological samples. Jingmeng Ju, Yan Liu and Min Xia interpreted the results. Yan Liu and Min Xia obtained funding and supervised this study. All authors made substantial contributions to the intellectual content of the paper and approved the final version of the manuscript.

## CONFLICT OF INTEREST STATEMENT

The authors declare no conflicts of interest.

## ETHICS STATEMENT

The study was approved by the Ethics Committee of School of Public Health, Sun Yat‐Sen University (2017‐001), and was in accordance with the principles of the Declaration of Helsinki. Written informed consents were obtained from each individual.

## Supporting information



Supporting Information

## Data Availability

Metagenomic sequencing data for all samples were deposited at NCBI Sequencing Read Archive (SRA Accession: PRJNA1251223). Other data that support the findings of this study were available from the corresponding authors upon reasonable request.
