# Supplementary material for: Microbial metabolism mediates the deteriorative effects of sedentary behaviour on insulin resistance
Source: Clin Transl Med. 2025 May 24;15(5):e70348. doi: 10.1002/ctm2.70348 (PMC12103651; doi:10.1002/ctm2.70348)
Supplement: Supplementary file 1 — Supporting Information [file CTM2-15-e70348-s001.docx]

**ADDITIONAL FILE TO**

**Microbial Metabolism Mediates the Deteriorative Effects of**

**Sedentary Behavior on Insulin Resistance**

Jingmeng Ju^1^, Jialin He^1^, Bingqi Ye^2^, Siqi Li^1^, Jiaqi Zhao^1^, Wanlan Chen^1^, Qi Zhang^1^, Wanying Zhao^1^, Jialu Yang^1^, Ludi Liu^2^, Yi Li^1^, Min Xia^1,*^ Yan Liu^1,*^

^1^Guangdong Provincial Key Laboratory of Food, Nutrition and Health, and Department of Nutrition, School of Public Health, Sun Yat-sen University, Guangzhou, Guangdong, 510080, P.R. China

^2^Guangdong Provincial Key Laboratory of Food, Nutrition and Health, and Department of Statistics and Epidemiology, School of Public Health, Sun Yat-sen University, Guangzhou, Guangdong, 510080, P.R. China

^*^Co-corresponding author

**Address for Correspondence**: Dr. Yan Liu, Department of Nutrition, School of Public Health, Sun Yat-sen University, Email: [liuyan215@mail.sysu.edu.cn](mailto:liuyan215@mail.sysu.edu.cn). OR Prof. Min Xia, Department of Nutrition, School of Public Health, Sun Yat-sen University, Email: [xiamin@mail.sysu.edu.cn](mailto:xiamin@mail.sysu.edu.cn).

Table of contents

[Supplementary Methods 3](#_Toc197353717)

[References 12](#_Toc197353718)

[Acknowledgments 13](#_Toc197353719)

[Supplementary Results 14](#_Toc197353720)

[Figure S1. Mendelian randomization estimates for the association between selected species and HOMA-IR. 14](#_Toc197353721)

[Figure S2. Leave-one-out plots for Mendelian randomization results 17](#_Toc197353722)

[Figure S3. Volcano plots of metabolites associated with sedentary time and HOMA-IR 19](#_Toc197353723)

[Figure S4. Metabolites mediating the causal association between microbial species and HOMA-IR. 21](#_Toc197353724)

[Figure S5. Distribution of intermediate metabolites and key enzymes in the microbial production of L-citrulline and L-serine. 22](#_Toc197353725)

[Figure S6. Sensitivity analysis of Mendelian randomization 24](#_Toc197353726)

[Table S1. Microbial species significantly associated with sedentary time and their affiliated phyla 25](#_Toc197353727)

[Table S2. Functional classification of KEGG pathways associated with at least three of the four identified species 27](#_Toc197353728)

# Supplementary Methods

**Inclusion and exclusion criteria of SCC-deep**

*Inclusion criteria*: (1) male participants aged between 35 to 74 years old; (2) absence of severe disability, any malignant tumors, thyroid disorder, biliary acute or chronic viral hepatitis, cirrhosis, chronic renal insufficiency, acute or chronic inflammatory disease; (3) absence of any gastrointestinal diseases, and (4) able to understand the nature and possible consequence of the study.

*Exclusion criteria*: (1) diagnosed diabetes, autoimmune diseases and heart diseases; (2) use of probiotics or other dietary supplements and medications known to influence gut microbiota within 3 months prior to sample collection.

**Collection of covariates and biological samples**

Information on demographic characteristics, medical history, drug use, lifestyles, and prevalence of comorbidities were collected with structured questionnaires by trained staffs. The International Physical Activity Questionnaire-Short-Form was used to collect physical activity over the past 7 days.^1^ Physical activity intensity was evaluated by metabolic equivalents as previously described.^2^ Smoking status was defined as self-reported tobacco use, and categorized as never, former or current smoker as previously described.^3^ Drinking was defined as the consumption of alcoholic beverages at least once a week during the past year.^4^ Diet information was collected using a validated Food Frequency Questionnaire, and a diet diversity score derived from 9 groups of foods, including cereals, vegetables, fruits, legumes, nuts, milk and dairy products, meat, eggs, and fish, was calculated. The intake frequency of each food group was measured on a 5-point scale, i.e., “almost every day”, “at least once per week”, “at least once per month”, “occasionally”, and “rarely or never”. If the response for one food group was “almost every day” or “at least once per week”, then one point was given, otherwise no point was given. Therefore, the diet diversity score ranged from 0 to 9, with higher scores representing greater diet diversity.^5^ In addition, socioeconomic factors were assessed via structured questionnaires. Education level was categorized into three groups: primary or below, middle school, and college or above. Household annual income was self-reported and classified into four predefined groups: <60,000, 60,000-100,000, 100,000-150,000, and ≥150,000 Yuan (RMB)/year.

Physical examinations and anthropometric measurements, including height, weight, BMI, waist circumference, and blood pressure were performed by trained staffs. Blood pressure was measured twice on the right upper arm in the sitting position after at least 5 min of rest using a validated digital automatic analyzer (Omron HEM-7136), and the average level was used.

Plasma samples following an overnight fast of 10 hours were collected and analyzed immediately at local laboratory. Plasma glucose, triglyceride, total cholesterol, high-density lipoprotein cholesterol (HDL-c), low-density lipoprotein cholesterol (LDL-c) were assessed by enzymatic methods on a Microplate Reader (Mindray BS800M; Mindray, Shenzhen, China). Insulin levels were measured with commercial ELISA kits (Mercodia, Uppsala, Sweden).

**Fecal DNA extraction, sequencing and Metagenomics analyses**

Participants received MGIEasy stool collection kit containing a room temperature stabilizing reagent and detailed instructions at the community center during the study visit. Stool samples were collected on the same day as blood drawing and stored at -20°C at the community center for a maximum of one day before transportation to the central freezer at -80°C until analysis. Stool DNA was extracted using the MagMAX^TM^ Microbiome Ultra Nucleic Acid Isolation Kit (Thermo Fisher Scientific, MA, USA). All samples were sequenced on the Illumina NovaSeq 6000 platform (Illumina, San Diego, California, USA; Paired-end; insert size, 350 bp; read length, 150 bp) by Novogene Co., Ltd (Beijing, China). The trimming and filtering of adaptors in metagenomics data were performed by fastp.^6^ Human reads were removed by aligning to the hg38 reference genomes with bowtie2 (v2.4.1, default parameters; coverage >80% of read).^7^ On average, 55.3 million high-quality reads per sample were generated for further analysis. Taxonomic profiling was performed using MetaPhlAn3 (v3.0.13),^8^ and clean reads were assembled into contigs with Megahit v1.2.9.^9^ Moreover, functional annotation to Kyoto Encyclopedia of Genes and Genomes (KEGG) Orthology (KO) was performed by Kofamscan v1.2.0.^10^ All taxa, KOs, and pathways data were reported as relative abundance (total sum scaling normalization).

**Selection of microbial species associated with sedentary time and HOMA-IR**

Alpha diversity in each sample at genus level was determined by R package *vegan* (V2.6.6.1). Community diversity between samples was evaluated by principal coordinates analysis (PCoA) based on Bray-Curtis distance and PERMANOVA analysis with the adonis function. After filtering out low-prevalence microbiome features (defined as a detection rate less than 5% for species and 5% for metabolic functions in all subjects), Spearman correlation was initially applied to identify significant associations between species and sedentary time (FDR *P*<0.05). Subsequently, a feature selection strategy was employed using the “random forest” algorithm with the R package *Boruta* (V8.0.0, 1000 trees, *P* value<0.05) to identify key species linked to sedentary time. Given that *Boruta* only provided feature importance without directions of the association, the directions of the association with sedentary time were determined from the Spearman correlation analysis. Moreover, the effect size of each key species derived from the Boruta analysis was used to construct a microbial score in an additive manner, reflecting the overall effect of key microbial species on sedentary time. To elucidate the potential mediation of sedentary time-associated species on the impact of sedentary time on HOMA-IR, causal mediation analysis with the R package *mediation* (V4.5.0) was conducted. The model was adjusted for demographics (age, BMI), lifestyle factors (smoking, drinking, and diet diversity), and socioeconomic factors (educational attainment and household income), and the robustness of the results was confirmed through a simulation exercise with 1000 bootstrapped replicates. Furthermore, given the zero-inflated nature of microbiome data, a two-part model^11^ accounting for both binary (present or not) and quantitative (relative abundance) features was applied to identify core species that were significantly associated with HOMA-IR, after adjustment for demographics, lifestyle, and socioeconomic factors.

**Human genomics and bi-directional** **Mendelian Randomization analysis**

Host DNA was extracted from buffy coat using TIANamp Blood DNA Kit from TIANGEN Co., Ltd (Beijing, China) according to the manufacturer’s instructions. The dosage of DNA used for subsequent library preparation was more than 1 μg and their concentration was controlled at no less than 80 ng/μL. Genotyping was performed with Infinium Chinese Genotyping Array-24 v1.0 BeadChip on the Illumina platform by WeGene Co., Ltd (Shenzhen, China). Quality control was then performed with PLINK (v.1.9), and single-nucleotide polymorphisms (SNPs) with (1) Minor Allele Frequency less than 5%; (2) Hardy-Weinberg equilibrium violation with *P*<1×10^-5^; and (3) a genotype calling rate below 5% were excluded^12^. Furthermore, we calculated linkage disequilibrium (LD) between each pair of SNPs at a window of 50 SNPs, and removed one of a pair of SNPs if LD exceeded 0.5. Finally, a total of 402,077 SNPs were retained for the genome-wide association study, among 554 participants. The threshold of *P*<5×10^-5^ was set for identifying SNPs associated with microbiota or metabolites to maximize the amount of genetic variance explained by selected SNPs, as previously described^12^.

To investigate the causal associations between selected microbial species and microbial metabolites on the variation of HOMA-IR, bi-directional MR analysis was performed using the *MendelianRandomization* R package (version 0.10.0). SNPs with F statistic (beta^2^/SE^2^) > 10 were considered as strong genetic IVs and were included in the subsequent MR analyses. MR estimates were calculated using various methods, including IVW, MR-Egger regression, and weighted median, with the IVW method serving as the primary method in our MR analyses. The 95% confidence intervals (CIs) for all MR methods were determined by the same statistical approach within the *MendelianRandomization* R package, based on standard normal approximation using the formula:

95%CI = $\hat{\beta}$ ±1.96×SE

To ensure the validity of the results, potential causal associations were identified based on the following criteria: (1) *P*<0.05 was obtained from the IVW method; (2) the effect sizes estimated by IVW, MR-Egger, and weighted median methods were consistent in direction; (3) *P*>0.05 for the intercept term calculated by the MR-Egger method, indicating no pleiotropy; and (4) *P*>0.05 for Cochran’s Q-test, indicating no heterogeneity. In addition, potential heterogeneous SNPs were identified through the “leave-one-out” analysis, which was performed by omitting each instrumental SNP in turn. Additionally, we performed reverse MR analyses on core species and metabolites following the same analysis procedure to validate the directionality of causality.

**Selection of microbial functions significantly associated with causal species**

Generalized linear regression was employed to identify individual microbial functions significantly associated with the four species causally related to HOMA-IR after adjustment for demographics, lifestyle, and socioeconomic factors. 1000× permutation tests were applied to control multiple comparisons. Functions significantly associated with at least three of the four causal species were regarded as important and subjected to further analysis. The effect sizes of the selected functions were then used to construct a microbial score as described above to elucidate the potential mediation of sedentary time-associated functions on the impact of sedentary time on HOMA-IR.

**Plasma** **metabolomics profiling and data analysis**

***Metabolomics profiling in plasma samples***

A total of 50 μL plasma samples were thawed on ice, vortexed for 10 sec, followed by the addition of 300 μL of pure methanol with internal standards, including L-2-chlorophenylalanine, [^2^H_3_]-L-carnitine HCl, 4-fluoro-L-α-phenylglycine, L-phenylalanine (2-^13^C, 99%), [^2^H_5_]-hippuric acid, [^2^H_5_]-kynurenic acid, [^2^H_5_]-phenoxy acetic acid. The mixture of plasma and internal standards was vortexed for 3 min, and the supernatants were recovered by centrifugation at 12,000 rpm for 10 min at 4°C. Then, 10 μL of the supernatant taken from each sample was pooled together to create a “mixed sample”, followed by a full scan mode with QTRAP^®^ 6500+ system to acquire a high-resolution data including RT, Q_1_, and Q_3_. Peak annotation of the mixed sample was performed with the in-house database of Metware Co., Ltd (Wuhan, China), followed by a transfer of the ion pair information to the triple quadrupole (QQQ) instrument. Finally, individual plasma samples were analyzed by QQQ in multiple reaction monitoring (MRM) mode to get a more accurate quantification of metabolites. More details for this process and instrument setup were as follows: chromatographic separation was performed on ACQUITY UPLC HSS T3 C18 (1.8 µm, 2.1 mm×100 mm, Waters) using an UPLC system (Shim-pack UFLC SHI-MADZU CBM A system, https://www.shimadzu.com/; QTRAP^®^ 6500+ System, https://sciex.com/). Mobile phase A was 0.1% formic acid in water, and mobile phase B was 0.1% formic acid in acetonitrile. Mass spectrometry (MS) detection was performed by triple quadrupole-linear ion trap mass spectrometry (QTRAP), equipped with an electrospray ionization (ESI) source. In chromatographic separation, 2 µL of sample was used and the flow rate was set at 0.35 mL/min. Full scans with a range of m/z 50-1000 were acquired in LIT and QQQ scans with positive and negative ion modes and controlled by Analyst 1.6.3 software (Sciex). The ESI source parameters were as follows: source temperature 500°C; ion spray voltage (IS) 5500 V (positive), -4500 V (negative); ion source gas I (GSI), gas II (GSII), curtain gas (CUR) was set at 55, 60, and 25.0 psi, respectively; the collision gas (CAD) was high. Instrument tuning and mass calibration were performed with 10 and 100 μmol/L polypropylene glycol solutions in QQQ and LIT modes, respectively. A specific set of MRM transitions were monitored for each period according to the metabolites eluted within this period.

***Metabolomics analyses***

For metabolomics data, we implemented a log transformation followed by Z-score scaling and normalization to address the extremely skewed distribution. Generalized linear regression was employed to identify individual metabolites significantly associated with both sedentary time and HOMA-IR, adjusted for potential confounders, including age, BMI, smoking, drinking, diet diversity, educational attainment, and household income. Moreover, mediation analysis was conducted to determine whether and to which extent the identified metabolites mediate the effect of the four causal species on HOMA-IR. 1000× permutation tests were applied to control for multiple comparisons. These results were visualized using Sankey diagrams, where the color and thickness of the lines indicated the direction and absolute value of the associations determined by generalized linear regression coefficients. Moreover, the associations between selected metabolites and HOMA-IR were further validated in an independent cohort recruited from Guangzhou (Luo et al.^13^), using general linear regression.

***In silico analysis for the downstream targets of identified metabolites***

The potential downstream targets of identified metabolites, including L-citrulline and L-serine, which could mediate the effect of causal species on HOMA-IR, were predicted using Super-PRED (<https://bioinformatics.charite.de/superpred>, with probability>80% and prediction accuracy>70%) and SwissTargetPrediction (<http://www.swisstargetprediction.ch/>). Furthermore, Gene Ontology enrichment analysis was then performed on the predicted downstream targets as input gene sets using the R package *clusterProfiler* (V4.12.0).^14^ The *simplifyEnrichment* (V1.14.0)^15^ package was then used to cluster the similarity matrices of the enriched terms into groups using the "kmeans" method.

***Identification of microbial enzymes involved in the production of selected metabolites***

The enzymatic functions and metabolic pathway annotations were retrieved directly from the KEGG database, a well-established knowledge base for metabolic and enzymatic functions. To elucidate how *Firmicutes bacterium CAG:83* and *Roseburia sp. CAG:471* influence the levels of L-citrulline and L-serine, a BLASTP search was performed using the protein sequences of enzymes known to be involved in the biosynthesis of these metabolites. The search was performed against all open reading frames of the two species. The presence of these enzymes (*argH, rocF* and *argF*, three enzymes responsible for the conversion of L-argininosuccinate to L-citrulline; and *lysC*, *asd*, *hom*, *thrB,* *thrC, ltaE*, and *glyA*, seven enzymes responsible for the transformation from L-aspartic acid to L-serine) was analyzed using BLASTP, with a threshold of >30% amino acid identity and an expected value of <0.05.^16^ Differential KOs between subjects with higher or lower sedentary time were identified using the Wilcoxon rank-sum test.

# References

1. Craig CL, Marshall AL, Sjostrom M, et al. International physical activity questionnaire: 12-country reliability and validity. *Med Sci Sports Exerc*. 2003;35:1381-1395.

2. IPAQ group. Guidelines for data processing and analysis of the International Physical Activity Questionnaire (IPAQ)[EB/OL]. <http://www.ipaq.ki.se/scoring.html>

3. Yang G, Fan L, Tan J, et al. Smoking in China: findings of the 1996 National Prevalence Survey. *JAMA*. 1999;282:1247-53.

4. Millwood IY, Walters RG, Mei XW, et al. Conventional and genetic evidence on alcohol and vascular disease aetiology: a prospective study of 500 000 men and women in China. *Lancet*. 2019;393:1831-1842.

5. Yin Z, Fei Z, Qiu C, et al. Dietary Diversity and Cognitive Function among Elderly People: A Population-Based Study. *J Nutr Health Aging*. 2017;21:1089-1094.

6. Chen S, Zhou Y, Chen Y, Gu J. fastp: an ultra-fast all-in-one FASTQ preprocessor. *Bioinformatics*. 2018;34:i884-i890.

7. Langmead B, Salzberg SL. Fast gapped-read alignment with Bowtie 2. *Nat Methods*. 2012;9:357-9.

8. Segata N, Waldron L, Ballarini A, Narasimhan V, Jousson O, Huttenhower C. Metagenomic microbial community profiling using unique clade-specific marker genes. *Nat Methods*. 2012;9:811-4.

9. Li D, Liu CM, Luo R, Sadakane K, Lam TW. MEGAHIT: an ultra-fast single-node solution for large and complex metagenomics assembly via succinct de Bruijn graph. *Bioinformatics*. 2015;31:1674-6.

10. Aramaki T, Blanc-Mathieu R, Endo H, et al. KofamKOALA: KEGG Ortholog assignment based on profile HMM and adaptive score threshold. *Bioinformatics*. 2020;36:2251-2252.

11. Fu J, Bonder MJ, Cenit MC, et al. The Gut Microbiome Contributes to a Substantial Proportion of the Variation in Blood Lipids. *Circ Res*. 2015;117:817-24.

12. Boulund U, Bastos DM, Ferwerda B, et al. Gut microbiome associations with host genotype vary across ethnicities and potentially influence cardiometabolic traits. *Cell Host Microbe*. 2022;30:1464-1480 e6.

13. Luo S, Zhao Y, Zhu S, et al. Flavonifractor plautii Protects Against Elevated Arterial Stiffness. *Circ Res*. 2023;132:167-181.

14. Wu T, Hu E, Xu S, et al. clusterProfiler 4.0: A universal enrichment tool for interpreting omics data. *Innovation (Camb)*. 2021;2:100141.

15. Gu Z, Hubschmann D. simplifyEnrichment: A Bioconductor Package for Clustering and Visualizing Functional Enrichment Results. *Genomics Proteomics Bioinformatics*. 2023;21:190-202.

16. Lopez-Bujanda ZA, Haffner MC, Chaimowitz MG, et al. Castration-mediated IL-8 promotes myeloid infiltration and prostate cancer progression. *Nat Cancer*. 2021;2:803-818.

# Acknowledgments

The authors would like to thank all study participants and the staffs in clinics for their help with the collection of biological samples. This work was supported by National Key Research and Development Program of China (2023YFC3606300), Key Project of National Natural Science Foundation of China (No. 82330105), Distinguished Young Scholars of the National Natural Science Foundation of China (Overseas, 21HAA01094), and Guangzhou Science and Technology Project (2024A04J6477).

# Supplementary Results

**Figure S1**


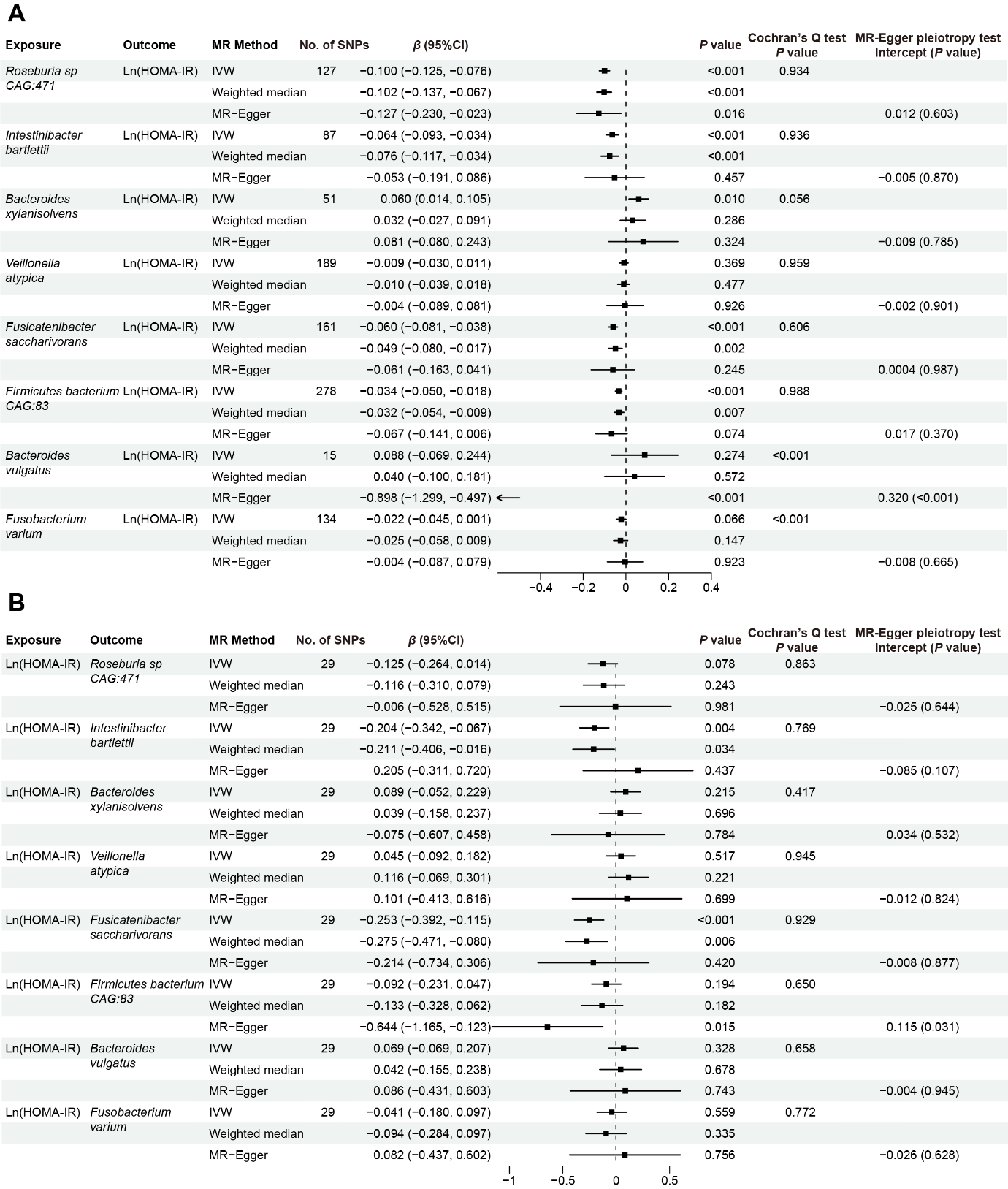


Figure S1. Mendelian randomization estimates for the association between selected species and HOMA-IR. (A) Mendelian randomization estimates for the association from selected species to HOMA-IR. (B) Mendelian randomization estimates for the association from HOMA-IR to selected species. IVW, inverse-variance-weighted.


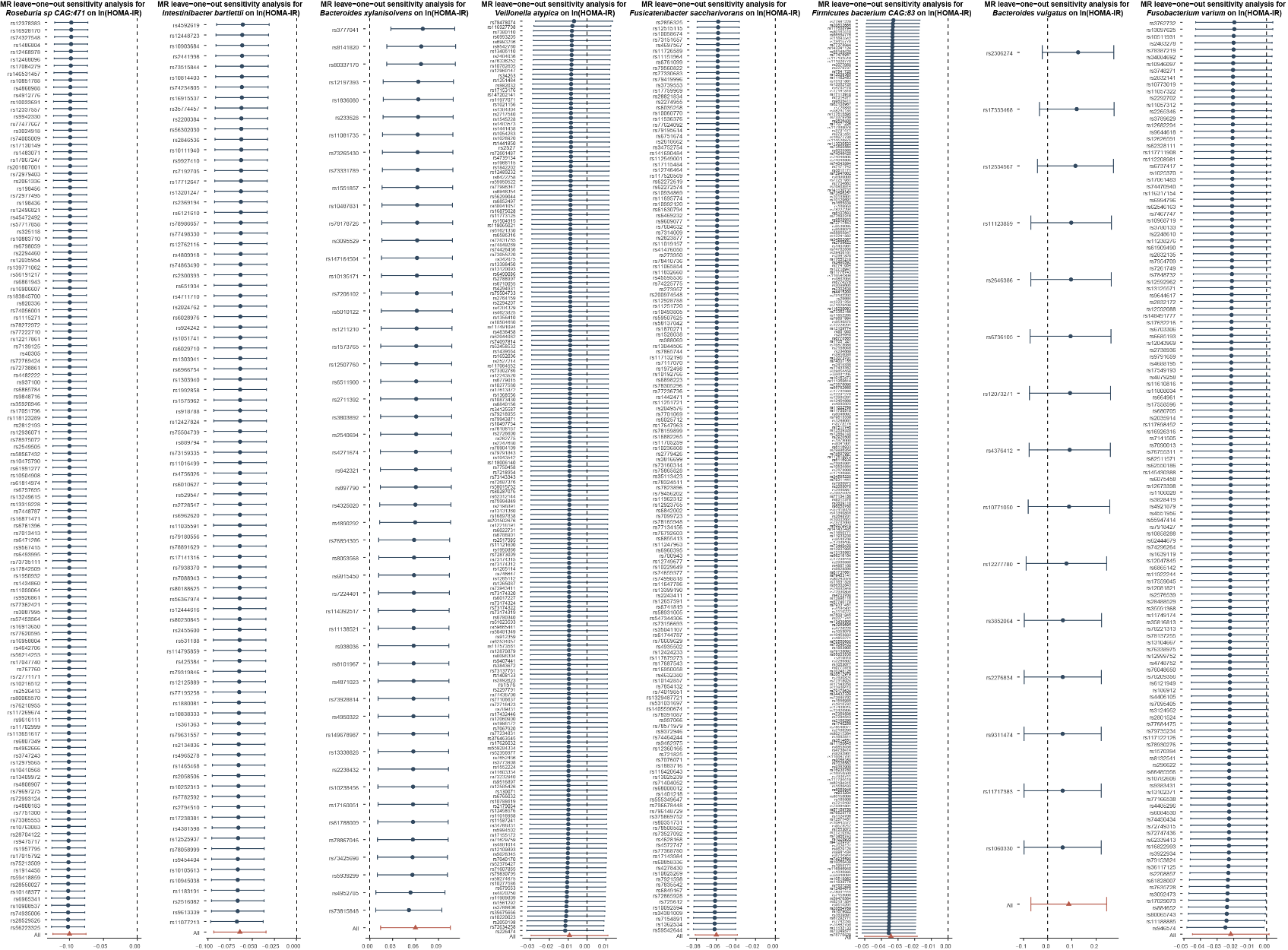
**Figure S2**

Figure S2. Leave-one-out plots for Mendelian randomization results**.** Each row represents the SNP-exposure effect size with corresponding standard error (SE). The red lines represent the average effect of all SNPs as calculated by the inverse variance weighted method.

**Figure S3**


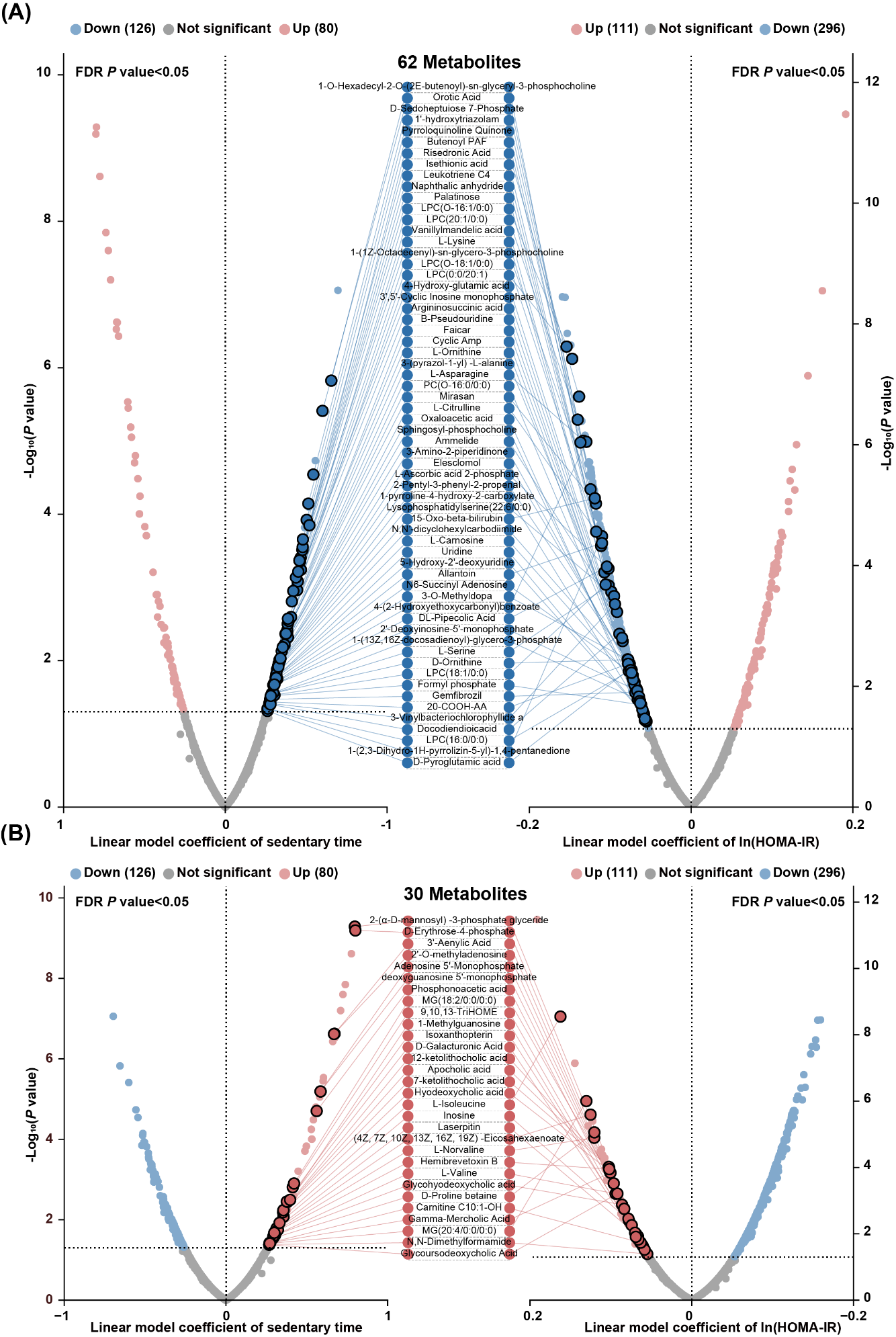


Figure S3. Volcano plots of metabolites associated with sedentary time and HOMA-IR**.** Generalized linear model was used to determine the associations of metabolites with sedentary time and HOMA-IR, adjusted for age, BMI, smoking, drinking, diet diversity, educational attainment, and household income. (A) Metabolites negatively associated with both sedentary time and HOMA-IR. (B) Metabolites positively associated with both sedentary time and HOMA-IR.

**FigureS4**

**
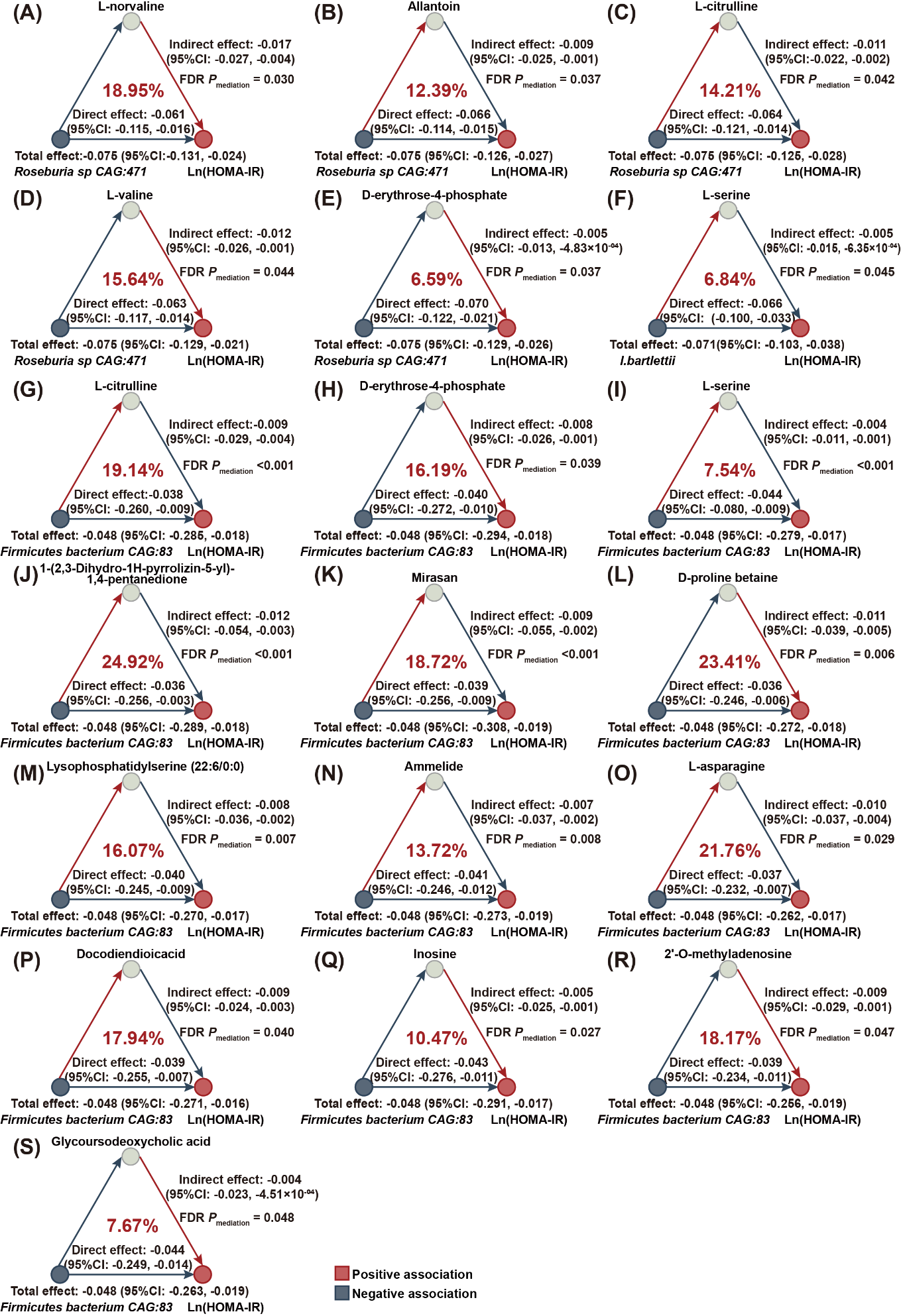
**

Figure S4. Metabolites mediating the causal association between microbial species and HOMA-IR. (A-E) Mediation effects of (A) L-norvaline, (B) Allantoin, (C) L-citrulline, (D) L-valine and (E) D-erythrose-4-phosphate on the associations between *Roseburia sp CAG:471* and HOMA-IR. (F) Mediation effect of L-serine on the associations between *Intestinibacter bartlettii* and HOMA-IR. (G-S) Mediation effects of (G) L-citrulline, (H) D-erythrose-4-phosphate, (I) L-serine, (J) 1-(2,3-Dihydro-1H-pyrrolizin-5-yl)-1,4-pentanedione (K) Mirasan, (L) D-proline betaine, (M) Lysophosphatidylserine (22:6/0:0), (N) Ammelide, (O) L-asparagine, (P) Docodiendioicacid, (Q) Inosine, (R) 2'-O-methyladenosine and (S) Glycoursodeoxycholic acid on the associations between *Firmicutes bacterium CAG:83* and HOMA-IR. All the mediation models were adjusted for age, BMI, smoking, drinking, diet diversity, educational attainment, and household income.

**Figure S5**


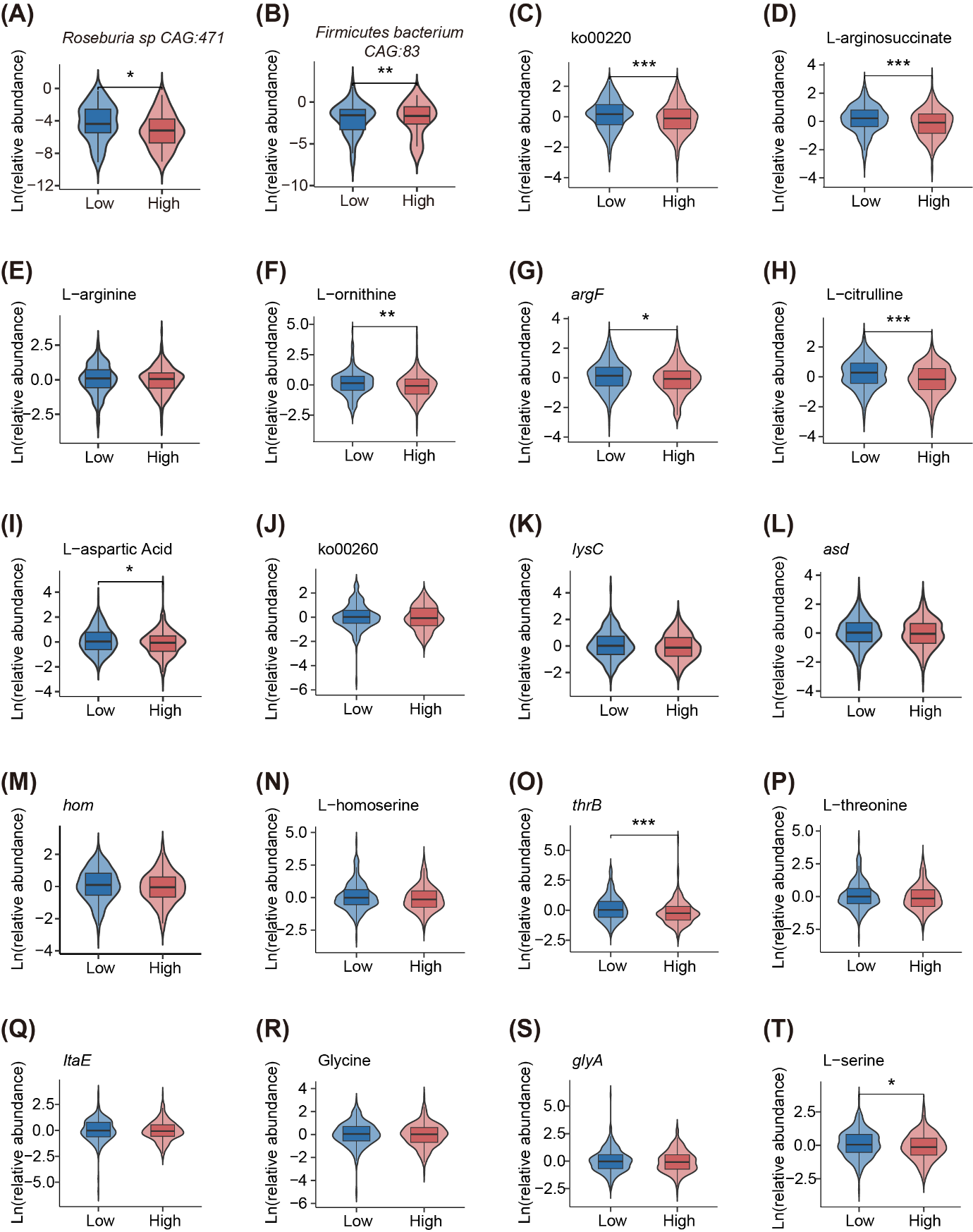


Figure S5. Distribution of intermediate metabolites and key enzymes in the microbial production of L-citrulline and L-serine. Distribution of (A) *Roseburia* *sp CAG:471*, (B) *Firmicutes bacterium CAG:83*, (C) ko00220, (D) L-arginosuccinate, (E) L-arginine, (F) L-ornithine, (G) *argF*, (H) L-citrulline, (I) L-aspartic acid, (J) ko00260, (K) *lysC*, (L) *asd*, (M) *hom*, (N) L−homoserine, (O) *thrB*, (P) L-threonine, (Q) *ltaE*, (R) Glycine, (S) *glyA* and (T) L-serine in subjects with longer or shorter sedentary time. *P* values were determined by Wilcoxon rank-sum test, with ^*^*P*<0.05, ^**^*P*<0.01, ^***^*P*<0.001.

**Figure S6**


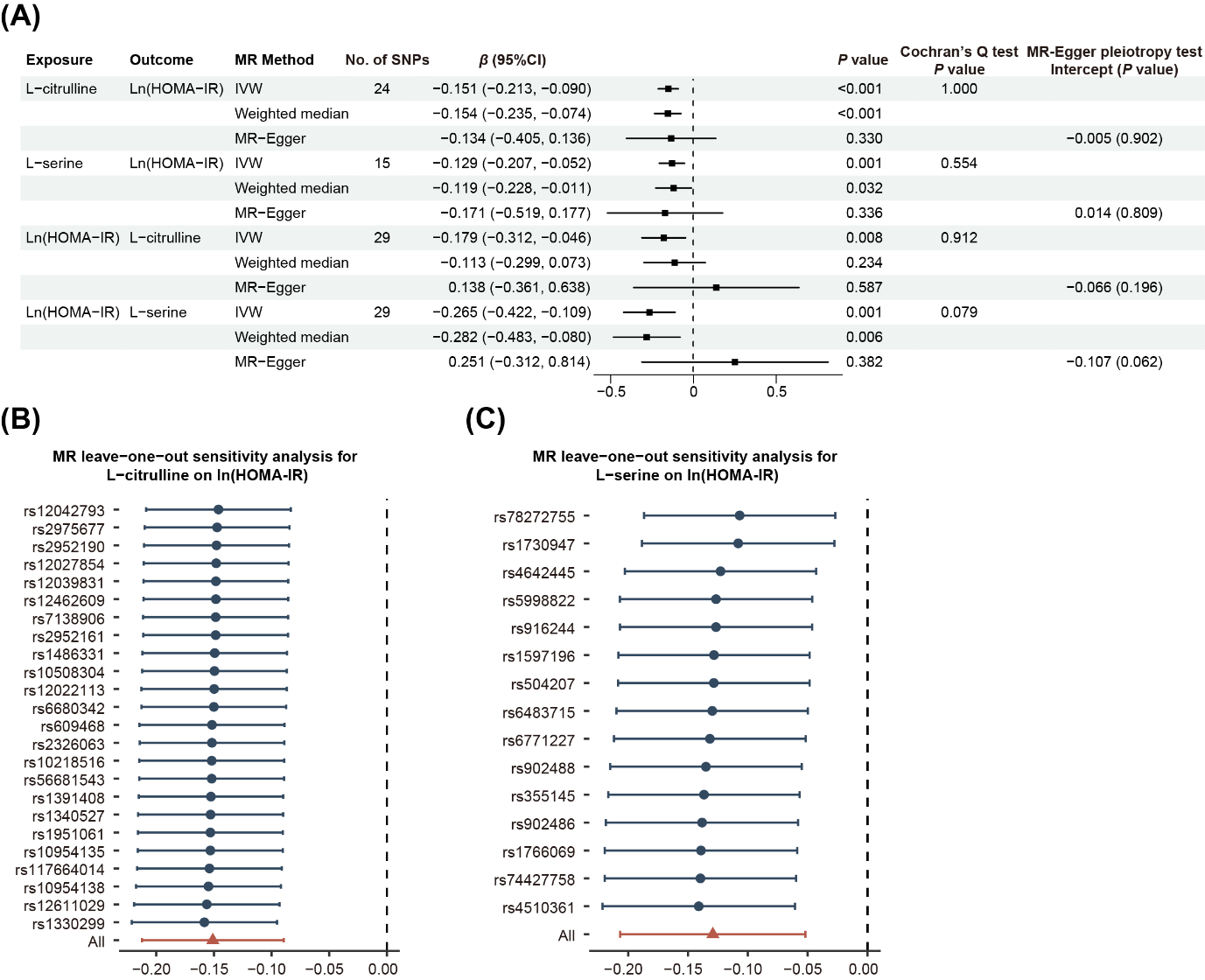


Figure S6. Sensitivity analysis of Mendelian randomization**.** (A) Mendelian randomization estimates for the association between selected metabolites and HOMA-IR. (B-C) Leave-one-out analysis for (B) L-citrulline and (C) L-serine. Each row represents the SNP-exposure effect size with the corresponding SE. The red lines represent the average effect of all SNPs as calculated by the inverse variance weighted method. IVW, inverse-variance-weighted.

# **Table S1.** Microbial species significantly associated with sedentary time and their affiliated phyla

| **No.** | **Species** | **Phyla** |
| --- | --- | --- |
| 1 | *Actinomyces_naeslundii* | *Actinomycetota* |
| 2 | *Actinomyces_oris* | *Actinomycetota* |
| 3 | *Actinomyces_sp_ICM47* | *Actinomycetota* |
| 4 | *Actinomyces_sp_oral_taxon_181* | *Actinomycetota* |
| 5 | *Actinomyces_sp_oral_taxon_414* | *Actinomycetota* |
| 6 | *Actinomyces_sp_S6_Spd3* | *Actinomycetota* |
| 7 | *Adlercreutzia_equolifaciens* | *Actinomycetota* |
| 8 | *Agathobaculum_butyriciproducens* | *Bacillota* |
| 9 | *Asaccharobacter_celatus* | *Actinomycetota* |
| 10 | *Bacteroides_ovatus* | *Bacteroidota* |
| 11 | *Bacteroides_thetaiotaomicron* | *Bacteroidota* |
| 12 | *Bacteroides_uniformis* | *Bacteroidota* |
| 13 | *Bacteroides_vulgatus* | *Bacteroidota* |
| 14 | *Bacteroides_xylanisolvens* | *Bacteroidota* |
| 15 | *Bifidobacterium_dentium* | *Actinomycetota* |
| 16 | *Blautia_wexlerae* | *Bacillota* |
| 17 | *Clostridium_bolteae* | *Bacillota* |
| 18 | *Clostridium_leptum* | *Bacillota* |
| 19 | *Clostridium_perfringens* | *Bacillota* |
| 20 | *Collinsella_aerofaciens* | *Actinomycetota* |
| 21 | *Collinsella_intestinalis* | *Actinomycetota* |
| 22 | *Collinsella_stercoris* | *Actinomycetota* |
| 23 | *Coprococcus_eutactus* | *Bacillota* |
| 24 | *Dorea_formicigenerans* | *Bacillota* |
| 25 | *Dorea_longicatena* | *Bacillota* |
| 26 | *Eggerthella_lenta* | *Actinomycetota* |
| 27 | *Enterorhabdus_caecimuris* | *Actinomycetota* |
| 28 | *Escherichia_coli* | *Pseudomonadota* |
| 29 | *Eubacterium_hallii* | *Bacillota* |
| 30 | *Eubacterium_ramulus* | *Bacillota* |
| 31 | *Eubacterium_rectale* | *Bacillota* |
| 32 | *Firmicutes_bacterium_CAG_83* | *Bacillota* |
| 33 | *Fusicatenibacter_saccharivorans* | *Bacillota* |
| 34 | *Fusobacterium_varium* | *Fusobacteriota* |
| 35 | *Gordonibacter_pamelaeae* | *Actinomycetota* |
| 36 | *Holdemania_filiformis* | *Bacillota* |
| 37 | *Intestinibacter_bartlettii* | *Bacillota* |
| 38 | *Intestinimonas_butyriciproducens* | *Bacillota* |
| 39 | *Lactobacillus_mucosae* | *Bacillota* |
| 40 | *Lactobacillus_salivarius* | *Bacillota* |
| 41 | *Lawsonibacter_asaccharolyticus* | *Bacillota* |
| 42 | *Olsenella_scatoligenes* | *Actinomycetota* |
| 43 | *Parabacteroides_distasonis* | *Bacteroidota* |
| 44 | *Parabacteroides_gordonii* | *Bacteroidota* |
| 45 | *Phascolarctobacterium_faecium* | *Bacillota* |
| 46 | *Phascolarctobacterium_succinatutens* | *Bacillota* |
| 47 | *Proteobacteria_bacterium_CAG_139* | *Pseudomonadota* |
| 48 | *Roseburia_sp_CAG_471* | *Bacillota* |
| 49 | *Streptococcus_anginosus_group* | *Bacillota* |
| 50 | *Streptococcus_gordonii* | *Bacillota* |
| 51 | *Streptococcus_infantis* | *Bacillota* |
| 52 | *Streptococcus_parasanguinis* | *Bacillota* |
| 53 | *Streptococcus_salivarius* | *Bacillota* |
| 54 | *Veillonella_atypica* | *Bacillota* |
| 55 | *Veillonella_sp_T11011_6* | *Bacillota* |

# Table S2. Functional classification of KEGG pathways associated with at least three of the four identified species

| Pathway ID | Category (Level 1) | Subcategory (Level 2) | Pathway (Level 3) |
| --- | --- | --- | --- |
| ko00040 | Metabolism | Carbohydrate metabolism | Pentose and glucuronate interconversions |
| ko00051 | Metabolism | Carbohydrate metabolism | Fructose and mannose metabolism |
| ko00061 | Metabolism | Lipid metabolism | Fatty acid biosynthesis |
| ko00120 | Metabolism | Lipid metabolism | Primary bile acid biosynthesis |
| ko00121 | Metabolism | Lipid metabolism | Secondary bile acid biosynthesis |
| ko00220 | Metabolism | Amino acid metabolism | Arginine biosynthesis |
| ko00260 | Metabolism | Amino acid metabolism | Glycine, serine and threonine metabolism |
| ko00270 | Metabolism | Amino acid metabolism | Cysteine and methionine metabolism |
| ko00311 | Metabolism | Biosynthesis of other secondary metabolites | Penicillin and cephalosporin biosynthesis |
| ko00332 | Metabolism | Biosynthesis of other secondary metabolites | Carbapenem biosynthesis |
| ko00401 | Metabolism | Biosynthesis of other secondary metabolites | Novobiocin biosynthesis |
| ko00460 | Metabolism | Metabolism of other amino acids | Cyanoamino acid metabolism |
| ko00473 | Metabolism | Metabolism of other amino acids | D-Alanine metabolism |
| ko00511 | Metabolism | Glycan biosynthesis and metabolism | Other glycan degradation |
| ko00513 | Metabolism | Glycan biosynthesis and metabolism | Various types of N-glycan biosynthesis |
| ko00515 | Metabolism | Glycan biosynthesis and metabolism | Mannose type O-glycan biosynthesis |
| ko00520 | Metabolism | Carbohydrate metabolism | Amino sugar and nucleotide sugar metabolism |
| ko00521 | Metabolism | Biosynthesis of other secondary metabolites | Streptomycin biosynthesis |
| ko00525 | Metabolism | Biosynthesis of other secondary metabolites | Acarbose and validamycin biosynthesis |
| ko00531 | Metabolism | Glycan biosynthesis and metabolism | Glycosaminoglycan degradation |
| ko00536 | Brite Hierarchies | Protein families: signaling and cellular processes | Glycosaminoglycan binding proteins |
| ko00540 | Metabolism | Glycan biosynthesis and metabolism | Lipopolysaccharide biosynthesis |
| ko00541 | Metabolism | Glycan biosynthesis and metabolism | O-Antigen nucleotide sugar biosynthesis |
| ko00550 | Metabolism | Glycan biosynthesis and metabolism | Peptidoglycan biosynthesis |
| ko00561 | Metabolism | Lipid metabolism | Glycerolipid metabolism |
| ko00564 | Metabolism | Lipid metabolism | Glycerophospholipid metabolism |
| ko00600 | Metabolism | Lipid metabolism | Sphingolipid metabolism |
| ko00603 | Metabolism | Glycan biosynthesis and metabolism | Glycosphingolipid biosynthesis - globo and isoglobo series |
| ko00604 | Metabolism | Glycan biosynthesis and metabolism | Glycosphingolipid biosynthesis - ganglio series |
| ko00630 | Metabolism | Carbohydrate metabolism | Glyoxylate and dicarboxylate metabolism |
| ko00680 | Metabolism | Energy metabolism | Methane metabolism |
| ko00770 | Metabolism | Metabolism of cofactors and vitamins | Pantothenate and CoA biosynthesis |
| ko00785 | Metabolism | Metabolism of cofactors and vitamins | Lipoic acid metabolism |
| ko00941 | Metabolism | Biosynthesis of other secondary metabolites | Flavonoid biosynthesis |
| ko00945 | Metabolism | Biosynthesis of other secondary metabolites | Stilbenoid, diarylheptanoid and gingerol biosynthesis |
| ko01051 | Metabolism | Metabolism of terpenoids and polyketides | Biosynthesis of ansamycins |
| ko03110 | Brite Hierarchies | Protein families: genetic information processing | Chaperones and folding catalysts |
| ko04090 | Brite Hierarchies | Protein families: signaling and cellular processes | CD molecules |
